# Study of the In Vitro Digestion of Olive Oil Enriched or Not with Antioxidant Phenolic Compounds. Relationships between Bioaccessibility of Main Components of Different Oils and Their Composition

**DOI:** 10.3390/antiox9060543

**Published:** 2020-06-20

**Authors:** Jon Alberdi-Cedeño, María L. Ibargoitia, María D. Guillén

**Affiliations:** Food Technology, Faculty of Pharmacy, Lascaray Research Center, University of the Basque Country (UPV-EHU), Paseo de la Universidad nº 7, 01006 Vitoria-Gasteiz, Spain; jon.alberdi@ehu.eus (J.A.-C.); marialuisa.ibargoitia@ehu.eus (M.L.I.)

**Keywords:** lipolysis, oxidation phenolic compounds, antioxidant efficiency, *gamma*-tocopherol, hydroxytyrosol acetate, dodecyl gallate, olive oil minor components, corn oil, virgin flaxseed oil

## Abstract

The changes provoked by in vitro digestion in the lipids of olive oil enriched or not with different phenolic compounds were studied by proton nuclear magnetic resonance (^1^H NMR) and solid phase microextraction followed by gas chromatography/mass spectrometry (SPME-GC/MS). These changes were compared with those provoked in the lipids of corn oil and of virgin flaxseed oil submitted to the same digestive conditions. Lipolysis and oxidation were the two reactions under consideration. The bioaccessibility of main and minor components of olive oil, of phenolic compounds added, and of compounds formed as consequence of the oxidation, if any, were matters of attention. Enrichment of olive oil with antioxidant phenolic compounds does not affect the extent of lipolysis, but reduces the oxidation degree to minimum values or avoids it almost entirely. The in vitro bioaccessibility of nutritional and bioactive compounds was greater in the olive oil digestate than in those of other oils, whereas that of compounds formed in oxidation was minimal, if any. Very close quantitative relationships were found between the composition of the oils in main components and their in vitro bioaccessibility. These relationships, some of which have predictive value, can help to design lipid diets for different nutritional purposes.

## 1. Introduction

Digestion is a very complex process in which the main reactions provoke hydrolysis of proteins, carbohydrates and lipids to yield smaller building blocks, which may be absorbed through the intestinal wall. Furthermore, other secondary reactions, such as oxidation, Maillard reaction, and even esterification among others, can also be produced during digestion [1,2,3]. All of them make up this process, which is essential to cover human nutritional needs. 

Lipids are an important group of macronutrients which include many different compounds. Triglycerides are their main components, and edible oils are the principal food lipid. During oil digestion triglycerides are hydrolyzed to give smaller molecules, of which only fatty acids and monoglycerides can be absorbed. Lipolysis extent determines the yield of molecules derived from oil main components that are able to be absorbed. Knowledge of the factors that influence the lipolytic process is a subject of great importance in monitoring the digestive process and designing lipids and mixtures of lipids with other components to cover different nutritional needs [4]. In this context, it has been proved that tea polyphenols are able to inhibit pancreatic lipase activity, reducing gastrointestinal lipolysis [5] and the absorption of lipids, thus diminishing the nutritional value of the lipids ingested. Likewise, it has been reported that the lipolysis degree reached during in vitro digestion of some oils is related to the oil composition [6,7,8]. This means that both main and minor oil components influence lipolysis yield, as could be expected. If the above-mentioned relations between oil composition and lipolysis degree were known in depth, they could be used to design lipids which are able to bring about specific degrees of lipolysis and suitable bioaccessibility of oil main components for different nutritional needs.

In addition to lipolysis, lipid oxidation can also take place during digestion [9,10,11,12,13,14], leading to the formation of toxic compounds with detrimental effects on health. It may be expected that the extent of this reaction will not be the same for all kinds of oils and that it will be determined by the oxidative stability of the lipids involved as well as by the presence or absence of minor compounds capable of acting as antioxidants or of prooxidants during digestion. This subject is also of great importance and should be taken into account in digestion studies, because, in addition to generating undesirable toxic bioaccessible compounds, the most reactive oxidation compounds could also influence or interfere with the digestion process in turn.

When edible oils are submitted to digestion, the above-mentioned reactions can also affect minor oil components, and the nature and properties of these can also, in turn, influence the digestive process [11]. As is known, edible oils are vehicles for vitamins and bioactive compounds, and it is desirable that the bioaccessibility of these minor components will be as high as possible, for their potential health effects. 

In summary, in order to advance in the understanding of the in vitro digestion of edible oils and to achieve a broad view, both of the evolution of the process and of the bioaccessibility of the compounds involved, as many influential factors as possible should be taken into account.

In this context, the in vitro digestion of olive oil is tackled. This oil is made up of a mixture of olive refined oil and of olive extra virgin oil, and as consequence is much poorer in antioxidant components than the latter. To the best of our knowledge, the behaviour of this oil under in vitro digestion conditions has not been previously studied. The study will pay attention firstly to lipolysis extent and to the pattern produced as well as to the bioaccessibility of the oil main components estimated using ^1^H NMR spectroscopic data of the lipid extracts of the digestates. In order to have a complete view of this lipolytic process the results will be analyzed jointly with those of other oils such as corn oil and virgin flaxseed oil, these latter from previous studies [13,14]. Relationships between the composition of these oils in their main components and in vitro bioaccessibility will be studied in order to find quantitative models to explain these relationships if any. The interest of these potential quantitative relationships is considerable because, if sound, they could be used as tools to design mixtures of oils for specific nutritional needs. Likewise, there will be an analysis of the effect of the enrichment of olive oil with various concentrations of dodecyl gallate, hydroxytyrosol acetate and *gamma*-tocopherol on the bioaccessibility of the oil main components in order to evaluate if these phenolic compounds are able to inhibit lipase activity. Furthermore, oxidation extent, if any, during in vitro digestion of olive oil enriched, or not, with phenolic compounds will be evaluated and compared with that undergone by corn oil and virgin flaxseed oil. Monitoring of oxidation extent, if any, will be tackled by using two different techniques. First of all, ^1^H NMR will be used to evaluate differences in the concentration of unsaturated fatty acids and acyl groups, in oil and in the lipid extract of the digestate, due to oxidation reactions, and secondly to quantify potential oxidation compounds in the lipid extract of the digestates. In addition, the abundance of volatile oxidation markers will also be estimated by means of SPME-GC/MS to clarify and/or reinforce the oxidation extent results obtained by the first technique. Finally, the bioaccessibility of all minor compounds involved in the in vitro digestion of olive oil enriched or not with phenolic compounds will also be determined. These compounds include natural olive oil minor components, phenolic added compounds, and potentially compounds formed by oxidation if any. 

## 2. Materials and Methods 

### 2.1. Samples Subject of Study

The study was carried out with two different olive oils O_1_ and O_2_, of the same brand, acquired in a local supermarket. As already mentioned, olive oil is made up of a mixture of extra virgin olive oil and of refined olive oil. The composition of both oils in molar percentages of linolenic (Ln), linoleic (L), oleic (O) and saturated (S) acyl groups, is very similar (O_1_: Ln% = 0.6 ± 0.1, L% = 8.0 ± 0.4, O% = 75.5 ± 0.6, and S% = 15.8 ± 0.2; O_2_: Ln% = 0.7 ± 0.1, L% = 8.0 ± 0.1, O% = 75.1 ± 0.6, and S% = 16.2 ± 0.5). This was determined from ^1^H NMR spectral data as in previous studies [15,16]. Both olive oils also contain a small concentration of alkanals. These are the aldehydes with the lowest reactivity of all, which could have been produced by a lipoxygenase mediated oxidation of unsaturated acyl groups during the crushing and malaxation steps of olive oil production, contributing, in low concentrations, to the green odour of olive oils [17]. Nevertheless, as is well known, these compounds may be formed in the absence of these enzymes, under very varied oxidative conditions. These olive oils also contain squalene and sterols, detectable by ^1^H NMR spectroscopy, and a certain number of terpenes and sesquiterpenes, detectable by SPME-GS/MS. Nevertheless, both abundance and number of these compounds are much smaller in olive oil than in extra virgin olive oils [18,19]. Likewise, the content of polyphenols is very small in these olive oils and they are not detectable by ^1^H NMR spectroscopy either in the standard proton spectrum or in the spectrum acquired by using the NOESYGPPS experiment, which will be explained later [18].

Aliquots of olive oil O_1_ were enriched with two different concentrations either of dodecyl gallate DG (purity 98%, from Alfa Aesar., GmbH & Co KG, Germany) or of hydroxytyrosol acetate HTA (purity of 99.54%, from Seprox Biotech, Madrid, Spain). Likewise, aliquots of the olive oil O_2_ were enriched with different concentrations of *gamma*-tocopherol (γT) (purity ≥90%, Eisai Food & Chemical Co. Ltd., Tokyo, Japan). These compounds were chosen due to their differing number of phenolic groups, which may influence on their activity. The samples enriched with dodecyl gallate were named, O_1_DG_1_ (with an enrichment of 0.12 mmol DG/mol [FA+AG]_O_) and O_1_DG_2_ (with an enrichment of 1.36 mmol DG/mol [FA+AG]_O_). The samples enriched with hydroxytyrosol acetate, were named O_1_HTA_1_ (with an enrichment of 0.28 mmol HTA/mol [FA+AG]_O_) and O_1_HTA_2_ (with an enrichment of 2.53 mmol HTA/mol [FA+AG]_O_). Finally, the oil O_2_ samples enriched with different concentrations of *gamma*-tocopherol were named O_2_γT_1_ (with an enrichment of 0.11 mmol γT/mol [FA+AG]_O_), O_2_γT_2_ (with an enrichment of 1.17 mmol γT/mol [FA+AG]_O_) and O_2_γT_3_ (with an enrichment of 12.58 mmol γT/mol [FA+AG]_O_). These enrichment levels were set in function of the solubility of these compounds in the oil. Thus, the above concentrations were obtained in order to reach enrichment degrees near to 0.02% and 0.2% in weight for the three phenolic compounds and, in addition, near 2% in weight in the case of *gamma*-tocopherol due to its high solubility in oils. However, this latter level of enrichment was not possible for dodecyl gallate and hydroxytyrosol acetate because of their limited solubility in oils. All these samples were submitted to in vitro digestion.

### 2.2. Digestion Experiments

Aliquots (0.5 g) of the above-mentioned samples were digested by using a semi-static in vitro gastrointestinal digestion model developed by Versantvoort et al. (2005) [20]. This validated method was optimized, in order both to improve lipid digestion and to reach lipolysis levels of a similar order to in vivo digestion [21]. It has three stages, which simulate digestive processes in mouth, stomach, and small intestine, by sequentially adding the corresponding digestive juices (saliva, gastric juice, duodenal juice and bile), whose composition is given in Table S1 (see Supplementary Material). The digestive juices were prepared in the following way: the electrolyte solutions of the digestate juices were prepared the day before the in vitro digestion experiment and the enzymes were added just before starting the in vitro digestion. Once the digestive juices were prepared, they were heated to 37 ± 2 °C to start de digestion experiment. The first stage begins by adding 6 mL of saliva to the sample. After 5 min of incubation, 12 mL of gastric juice are added and the mixture is rotated head-over-heels at 40 rpm for 2 h at 37 ± 2 °C. One hour after the start of the gastric stage, pH is set between 2 and 3 with HCl (37%), simulating the gradual acidification of the chyme occurring in vivo. After 2 h of the gastric stage, 2 mL of sodium bicarbonate solution (1 M), 12 mL of duodenal juice, and 6 mL of bile juice are added. Subsequently, pH is set between 6 and 7, and the mixture is again rotated at 40 rpm and incubated at 37 ± 2 °C for 4 h. All the reagents and enzymes for the preparation of digestive juices were acquired from Sigma-Aldrich (St. Louis, MO, USA): α-amylase from *Aspergillus oryzae* (10065, ~30 U/mg); pepsin from porcine gastric mucosa (P7125, ≥400 U/mg protein); amano lipase A from *Aspergillus niger* (534781, ≥120,000 U/g); pancreatin from porcine pancreas (P1750); lipase type II crude from porcine pancreas (L3126, 100–500 U/mg protein (using olive oil, 30 min incubation); and bovine bile extract (B3883). The digested samples were named like the original samples preceded by D (DO_1_, DO_1_DG_1_, DO_1_DG_2_, DO_1_HTA_1_, DO_1_HTA_2_, DO_2_, DO_2_γT_1_, DO_2_γT_2_ and DO_2_γT_3_). Two digestion experiments, each including duplicate samples, were performed. Blank samples corresponding to the mixture of juices submitted to digestive conditions were also taken for further analysis.

### 2.3. Digestate Lipid Extraction

Lipids from the digestates were extracted using dichloromethane as solvent, added directly to the digestates without any previous step, (CH_2_Cl_2_, HPLC grade, Sigma-Aldrich) following a methodology that also allows fatty acid extraction as in previous studies [9]. This methodology involves a three-stage liquid-liquid extraction process with 20 mL of CH_2_Cl_2_ each. Afterwards, to ensure a complete protonation of fatty acids and/or the dissociation of the potential salts formed, the remaining water phase was acidified to pH 2 with HCl (37%) and a second extraction was carried out in three steps using again 20 mL of CH_2_Cl_2_. For this purpose, a Sigma 3K30 centrifugal machine (Sigma Laboratory Centrifuges, Germany) working at 2724 g was used, each extraction step lasting 6.50 min. All the CH_2_Cl_2_ extracts of each sample were mixed and the solvent was eliminated by means of a rotary evaporator under reduced pressure at room temperature, in order to avoid lipid oxidation. The extraction yield was in all cases near 85%. These extracts contain triglycerides, diglycerides and monoglycerides, as well as fatty acids and minor lipophilic compounds either present in the original samples or formed in the digestion process.

### 2.4. Study by ^1^H NMR of Oil Samples and Lipid Extracts of Digestates

#### 2.4.1. Samples Subject of Study and Operating Conditions

The ^1^H NMR spectra of the original oils **O_1_ and O_2_**, and of the oil samples enriched with each one of the phenolic compounds above mentioned at the different concentrations (O_1_DG_1_, O_1_DG_2_, O_1_HTA_1_, O_1_HTA_2_, O_2_γT_1_, O_2_γT_2_ and O_2_γT_3_), and of the lipids extracted from their digestates (DO_1_, DO_1_DG_1_, DO_1_DG_2_, DO_1_HTA_1_, DO_1_HTA_2_, DO_2_, DO_2_γT_1_, DO_2_γT_2_ and DO_2_γT_3_), were acquired in duplicate using a Bruker Avance 400 spectrometer operating at 400 MHz. As in previous studies [18,22] standard ^1^H NMR and multisuppressed spectra were acquired, these latter by using NOESYGPPS experiments. Although these latter experiments are capable of detecting phenolic compounds when they are in concentrations similar to those found in extra virgin olive oils [18], their sensitivity is not sufficient to detect them when they are in lower concentrations, as in the case of olive oils involved in this study. Details about operating conditions are given in Supplementary Material. 

#### 2.4.2. Identification of the Components from ^1^H NMR Spectral Data

**Figure 1:**
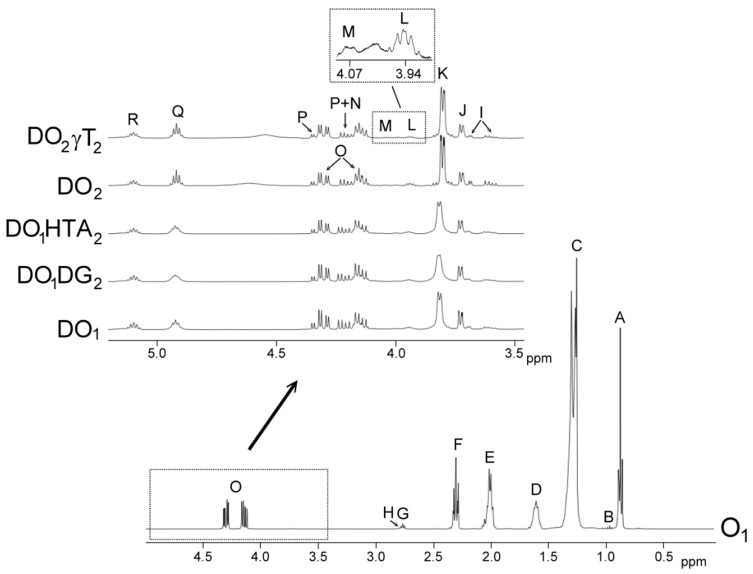
Region comprised between 0.0 and 4.9 ppm, of olive oil O_1_^1^H NMR spectrum, and region comprised between 3.5 ppm and 5.10 ppm, conveniently enlarged, of the ^1^H NMR spectra of the lipids extracted from the several digestates (DO_1_, DO_1_DG_2_, DO_1_HTA_2_, DO_2_ and DO_2_γT_2_), in which signals of protons of their main components appear. The signal letters agree with those of Tables S2 and S3 of Supplementary Material.

The identification of the components present in the original oils, in the oil samples enriched with phenolic compounds and in the lipid extracts of their digestates, was carried out on the basis of the assignments of the ^1^H NMR signals to the different kinds of hydrogen atoms, and in short to the different compounds. Figure 1 gives the spectral regions comprised between 0.0 and 4.9 ppm, of olive oil O_1_^1^H NMR spectrum, and between 3.5 ppm and 5.10 ppm, conveniently enlarged, of the ^1^H NMR spectra of the lipids extracted from the several digestates (DO_1_, DO_1_DG_2_, DO_1_HTA_2_, DO_2_, and DO_2_γT_2_), in which signals of protons of their main components appear. 

These above-mentioned signals, and others due to protons of minor components not shown in Figure 1, but present in the spectra of the above-mentioned samples, their chemical shifts and assignments are given in Supplementary Tables S2–S5. These assignments were made taken into account previous studies as is indicated in each table, or were based on the signals of standard compounds acquired for this study, which include cycloartenol, squalene, hexanal and decanal, acquired from Sigma-Aldrich (St. Louis, MO, USA) and linolein hydroperoxides purchased from Cayman Chemical (Ann Arbor, MI, USA).

Table S2 shows ^1^H NMR signals of specific protons of the different glyceride structures, such triglycerides, diglycerides and monoglycerides. Table S3 shows ^1^H NMR signals of protons of linolenic, linoleic, oleic and saturated acyl groups and fatty acids, and the signals of methylenic protons supported on carbons atoms in *alpha* and *beta* position in relation to carbonyl-carboxyl groups. Table S4 shows ^1^H NMR signals of protons of oxidation compounds coming from main oil component degradation, which occurred during digestion. Finally, Table S5 gives ^1^H NMR signals of some protons of dodecyl gallate, hydroxytyrosol acetate, *gamma*-tocopherol, of free and esterified cycloartenol plus 24-methylenecycloartenol and of squalene. The areas of some of these spectral signals were used to quantify the concentration of the different kinds of the above-mentioned structures in the corresponding samples, as will be explained below. 

#### 2.4.3. Quantifications Made from ^1^H NMR Spectral Data

This technique allows the estimation of the concentrations, expressed in different ways, of all identified compounds if they do not have overlapped signals in the corresponding spectra. This is possible because, as has been explained above, the area of the ^1^H NMR signals is proportional to the number of protons that generate the signal. The quantification of the different kinds of compounds or structures is explained below. 

##### Estimation of the Molar Percentage of the Different Kinds of Glycerides in the Digestates

This estimation can be carried out by using the intensity of some signals indicated in Supplementary Tables S2 and S3, which are also shown in Figure 1. Although glycerol is formed during digestion, due to its polar nature it is not present in the lipid extract of the digestate. However, its concentration can be estimated indirectly. This is possible because the concentration of total fatty acids plus acyl groups, of only acyl groups, and of fatty acids released in the formation of diglycerides and monoglycerides can be determined from ^1^H NMR data. Thus, the estimation of the molar percentage of triglycerides (TG), 1,2-diglycerides (1,2-DG), 1,3-diglycerides (1,3-DG), 2-monoglycerides (2-MG), 1-monoglycerides (1-MG), and glycerol (Gol) in relation to the total glyceryl structures present in the digestate, was carried out by using equations [eq. S1–eq. S10] given in Supplementary Material and the areas of signals included in Supplementary Tables S2 and S3. They are based exclusively on the intensity of ^1^H NMR spectral signals [23]. 

##### Estimation of the Molar Percentage of Fatty Acids Plus Acyl Groups That Have Linolenic, Linoleic, Oleic and Saturated Structures in Relation to the Total Fatty Acids and Acyl Groups in Digestates

In edible oils the concentration of fatty acids is very low and, in many cases, inappreciable in comparison with the concentration of acyl groups. However, as is known, during oils digestion hydrolysis provokes the transformation of a certain number of acyl groups into fatty acids. The fatty acids formed maintain the same number of carbon atoms and unsaturation pattern as the starting acyl groups. Acyl groups and fatty acids having the same structure provide NMR spectra signals with a high degree of overlapping that allow their joint quantification. In this study, the molar percentage *of linolenic, linoleic, oleic and saturated structures* found in acyl groups and fatty acids in the digestates was estimated in relation to the total number of moles of fatty acids plus acyl groups. This estimation was made using Supplementary Equations (S11)–(S14), in which the areas of some signals that are shown in Figure 1 and in Supplementary Table S3 are involved. These equations are the same as those employed in previous studies [15,16], but using the signal of methylenic protons supported on carbons atoms in *alpha* position in relation to carbonyl-carboxyl groups, instead of the signal of triglyceride protons used in edible oils studies. 

##### Estimation of the Concentration of Specific Compounds (x) in Oil Samples and in the Digestates

The concentration of oxidation compounds, and of others such as squalene, cycloartenol plus 24-methylenecycloartenol, dodecyl gallate, hydroxytyrosol acetate and *gamma*-tocopherol either in oils or in digestates can be estimated by using the general equation [eq. S15] given in Supplementary Material and the intensity of one of their non-overlapped ^1^H NMR spectral signals, which are indicated in Figure 1, and in Supplementary Tables S3–S5. This equation allows one to estimate the concentration of any compound in oils or in digestates in relation to the concentration of fatty acids plus acyl groups, which are considered the internal reference.

##### Estimation of In Vitro Bioaccessibility

The in vitro bioaccessibility of a compound can be defined as the concentration of the compound that remains absorbable after in vitro digestion. This concentration may refer either to an internal reference or to the initial concentration of the compound in the sample before digestion. The first approach is much more general because it can be used for compounds formed during digestion and absent in the sample before digestion. In this study the internal reference can be the concentration of oil main components expressed by the sum of the concentration of fatty acids plus acyl groups in the digestate ([FA]+[AG])_D_. In vitro bioaccessibility, thus defined, of the oil main components, can be estimated by using the equation B_OMC_ = ([FA]+[MG])_D_/([FA]+[AG])_D_, because the only absorbable compounds coming from oil main components are fatty acids, FA, and monoglycerides, MG. For any other compound X present in the oil sample before digestion or not, the equation to be used to determine the bioaccessibility in this approach is B_x_ = [X]_D_/([FA]+[AG])_D_, where [X]_D_ is the concentration of the compound X in the lipid extract of the digestate.

In the second approach, bioaccessibility B′ can be estimated by the ratio between the concentration of the compound in the lipid extract of the digestate [X]_D_ and the concentration in the oil before digestion, [X]_O_, as indicated in the equation B′_x_ = [X]_D_/[X]_O_. This definition gives information about the loss of the compounds during in vitro digestion, or about the fraction of the compounds released during digestion that are really absorbable as in the case of oil main components. For oil main components, B_OMC_ and B′_OMC_ are very similar because the reference is barely modified during digestion. 

### 2.5. Study by SPME-GC/MS of the Headspace of the Digestates and of the Mixture of the Digestive Juices Submitted to Digestion Conditions with Olive Oil 

Extraction of the volatile components constituting the headspace of the several samples (0.5 g in a 10 mL screw-cap vial) was accomplished automatically by using a CombiPAL autosampler (Agilent Technologies, Santa Clara, CA, USA). The samples studied were the several digestates (DO_1_, DO_1_DG_1_, DO_1_DG_2_, DO_1_HTA_1_, DO_1_HTA_2_, DO_2_, DO_2_γT_1_, DO_2_γT_2_, and DO_2_γT_3_) and the mixtures O_1_DJ and O_2_DJ of digestive juices DJ, after undergoing digestion conditions, and olive oils O_1_ and O_2_. The comparison of the headspaces of the several samples enables one to deduce differences provoked by in vitro digestion. The operating conditions were the same as those used in previous studies [24] and are explained in Supplementary Material.

Identification of the headspace components was carried out by using several commercial standard compounds acquired from Sigma-Aldrich (St. Louis, MO, USA). When standard compounds were not available, identification was made by matching the spectra obtained, higher than 85%, with those of commercial libraries (Wiley W9N08, Mass Spectral Database of the National Institute of Standards and Technology), or with those spectra provided by the scientific literature, as in previous studies [24].

The semi-quantification of the compounds was based on the area counts of the base peak (Bp) of the mass spectrum of each compound divided by 10^6^. When the Bp of a compound overlapped with some ion peak of the mass spectrum of another compound, an alternative ion peak was selected for the semi-quantification of the former [24]. Although the chromatographic response factor of each compound is different, the area counts thus determined are useful for the comparison of the abundance of each compound in the different samples. The target compounds of this technique were the volatile oxidation compounds formed in in vitro digestion, and terpenes and sesquiterpenes, which are characteristic minor volatile components of olive oil. Data given in the corresponding tables are average values of duplicate experiments. 

### 2.6. Statistical Analysis 

The significance of the differences among samples in the several kinds of data, was determined by one-way variance analysis (ANOVA) followed by Tukey *b* test at *p* < 0.05, using SPSS Statistics 24 software (IBM, NY, USA). 

## 3. Results

The main reaction that takes place during digestion is the hydrolysis of large molecules, such as proteins, triglycerides and carbohydrates, to release molecules of a small size capable of being absorbed through the intestinal wall. The extent and pattern of this reaction determines the bioaccessibility of these main components. Nevertheless, hydrolysis also could affect smaller molecules whenever they have hydrolyzable bonds. Furthermore, other reactions such as oxidation reactions could also be produced affecting both main and minor components, either present in or added to the food. These latter reactions could also give rise to the formation of derived compounds, some of which could also be absorbed, affecting the bioaccessibility of the different kinds of compounds. In this context the in vitro digestion of olive oil, enriched or not with phenolic compounds, will be addressed and compared with that of other oils of very different composition, such as corn and virgin linseed oils.

### 3.1. Lipolysis Extent and In Vitro Bioaccessibility of Oil Main Components of Olive Oil, Comparison with Those of Corn and Virgin Flaxseed Oil, and Effect of Olive Oil Enrichment with Phenolic Compounds

#### 3.1.1. Lipolysis and In Vitro Bioaccessibility of Olive Oil Main Components

Lipolysis provokes the release of fatty acids (FA) by breaking the ester bonds of triglycerides, yielding also diglycerides (DG), monoglycerides (MG) and glycerol (Gol). Table 1 gives the molar percentages of each of the glyceride structures present in the digestates formed during the digestion of two different olive oils, estimated as described in the experimental section. It can be observed that the main glyceryl lipolytic products formed in the digestion of both oils O_1_ and O_2_ are monoglycerides (near 44% and 42% in DO_1_ and DO_2_ respectively) and glycerol (near 28% and 26% in DO_1_ and DO_2_ respectively). This is very important because both fatty acids and monoglycerides are able to be absorbed through the intestinal wall. By contrast, the concentration of triglycerides in the digestates of both oils is low, reduced to near 13%, and that of diglycerides reaches near 15% and near 18% in DO_1_ and DO_2_ respectively. 

A parameter that represents both the extent and pattern of the lipolysis reached during in vitro digestion in a global way is the in vitro bioaccessibility of the oil main components, B_OMC_, defined as the ratio between the real absorbable molecules after digestion and all absorbable potential molecules before digestion. The really absorbable molecules after digestion are the released fatty acids (FA) and monoglycerides (MG), (FA+MG)_D_, present in the digestate. The potential absorbable molecules are fatty acids plus all acyl groups, (FA+AG)_D_. In agreement with the molar percentage of the different kinds of glyceryl structures, given in Table 1, B_OMC_ (determined as indicated in the experimental section) of DO_1_ is slightly higher than DO_2_. This parameter is very important because it not only summarizes in a single value the level of lipolysis reached during the in vitro digestion but also because of its nutritional meaning.

#### 3.1.2. Comparison between Lipolysis Yield of Olive Oil and In Vitro Bioaccessibility of Its Main Components and Those of Corn and Virgin Flaxseed Oils Submitted to the Same Digestive Conditions 

The data of lipolysis yields of other edible oils such as corn oil C and virgin flaxseed oil F, obtained in previous studies [13,14] under the same digestive conditions as in this study, are given in Table 1. It can be observed that the extent and pattern of lipolysis is very different to that of olive oils O_1_ and O_2_. The concentration of monoglycerides in the digestates of corn oil DC and of virgin flaxseed oil DF reaches values near 31% and 24% respectively, somewhat lower than that of the olive oils. These results are in agreement with previous studies in which important differences in the extent of the lipolysis reached during in vitro digestion of edible oils of different compositions were also found [6,7,8]. As expected, there is a clear difference between the bioaccessibility of oil main components in DF and in DC, DO_1_, or DO_2_, as Table 1 shows. Although the difference between the bioaccessibility of the main components of the oil in DC and in DO_1_ or DO_2_ is not statistically significant, the bioaccesibility in DC tends to be smaller than in DO_1_ and DO_2_. This is in line with the lower extent of the lipolysis undergone by C and F oils during in vitro digestion compared with that of olive oils, which is also reflected in the molar percentages of different glyceryl species of the corresponding digestates.

There may be many factors that influence the in vitro digestion lipolytic process. However, under the same digestive conditions, the minor and main oil components present can be considered the main ones. To the best of our knowledge, there are few studies regarding the influence of oil minor components. As one example, no significant differences have been found in the distribution of the glycerides in the digestates of refined and virgin soybean oils with different content in minor components [11]. The minor components of the three oils considered here are very different not only in their nature but also in their concentrations, which is why no conclusion could be drawn in this regard. It could only be mentioned that the three oils, as Table 1 shows, contain a small concentration of 1,2-diglycerides which can act from the beginning of the in vitro digestion process as emulsifiers, favouring contact between enzymes and lipid active sites to facilitate the lipolytic reactions. However, as the difference in the initial concentration of 1,2-diglycerides in olive, corn and virgin flaxseed oils is very small it is to be expected that this factor has no influence on the lipolysis extent produced in these oils during digestion.

As already mentioned, main oil components can also be determinant factors of the lipolysis extent during in vitro digestion. There are some studies on this issue [8,25,26,27,28,29]. The oil’s main components are triglycerides, which support different kinds of acyl groups, with varied number of carbon atoms and unsaturation degrees. Furthermore, the acyl groups can occupy different positions in the backbone of the glyceryl group, forming in this way different kinds of triglycerides. All these variables can influence the extent and pattern of lipolysis during in vitro digestion. 

##### Influence of the Length and Unsaturation Degree of Acyl Groups Present in the Oil

The acyl groups of the oils here considered differ in length and, as Table 2 shows, have important differences in the unsaturation degree. Furthermore, it should be mentioned that, in olive and corn oils, which have similar molar percentages of saturated acyl groups S, the distribution of these between palmitic and stearic groups is of a similar order in both oils, the second group in a much smaller percentage than the first, as is well known [30]. However, in virgin flaxseed oil the molar percentage of saturated acyl groups S is smaller than in the other two oils, having only a slightly smaller percentage of stearic than of palmitic groups [31]. Taking into account all the compositional data and lipolysis extent of each oil shown in Table 1, it seems evident that the unsaturation degree (or the saturation degree) of the oils greatly influences lipolysis extent reached during their digestion, in contrast to the reported in some previous studies [28,32]. As Table 1 and Table 2 show, the most unsaturated oil (virgin flaxseed oil), reaches the lowest lipolysis extent during digestion and the opposite is true for olive oils. Likewise, from data of these tables it is evident that oleic acyl group has a slightly greater tendency to be hydrolysed during digestion than linoleic acyl group. This is evident because olive and corn oils have similar molar percentages of saturated acyl groups, but olive oils, which are richer in oleic acyl groups, reach a lipolysis extent during digestion which is slightly higher than the second oil, which is richer in linoleic groups. This fact is in disagreement with the similar tendency of oleic, linoleic and even of linolenic acyl groups to hydrolyze reported by some authors [27]. Furthermore, it has also been described that ester bonds of saturated acyl groups, such as palmitic and stearic groups, are hydrolyzed more easily or faster by pancreatic lipase than unsaturated acyl groups such as oleic, linoleic and linolenic acyl groups [27,28,29]. In addition, it has also been reported that hydrolysis is more efficient the smaller the number of carbon atoms of the acyl groups [6,27,29,33]. For this reason, the tendency of palmitic group to hydrolyze should be greater than that of stearic group, although some authors find no difference between them [32,34,35]. Compositional data of the oils involved in this study given in Table 2 do not permit an analysis of some of the above-cited considerations. 

##### Influence of the Distribution of the Different Kinds of Acyl Groups in the Backbone of the Triglycerides in Each Oil

Some authors have also pointed out that, in addition to the above mentioned concerning different tendencies of fatty acyl groups to be hydrolyzed in function of their unsaturation degree and length, the positions which they occupy in the backbone of triglyceride could also influence the hydrolysis extent reached during their in vitro digestion. The importance of the distribution of the different acyl groups in triglyceride is due to the ester hydrolysis which takes place mainly in the sn-1 and sn-3 positions of the triglyceride when pancreatic lipase and *A. niger* lipase are used [8,25,29]. Due to this, the distribution of the different kinds of acyl groups in the backbone of the triglycerides of oils obtained by a similar processing and same vegetable origin as those involved in this study was analyzed using data from the literature [36,37]. Published data about triglyceride profiles of these three kinds of oils evidence that none of these oils have triglycerides with both sn-1 and sn-3 positions occupied simultaneously by saturated acyl group, which is considered the group most likely to be hydrolyzed [27,28,29]. Saturated acyl groups occupy, almost exclusively, the sn-1 position in the triglycerides of these three oils and the abundance of this class of triglycerides in each oil depends on the molar percentage of this type of acyl groups in the oil. As Table 2 shows, the molar percentage of saturated acyl groups is very similar in olive and corn oils and slightly less in virgin flaxseed oil. For this reason, the formation of 1,2-diglycerides as consequence of the hydrolysis of the ester group of saturated acyl groups should be of the same order in olive and corn and lower in virgin flaxseed oil. However, this does not explain the differences found in the total lipolysis extent undergone by these oils during in vitro digestion. 

Analysis of the profile of the triglycerides of these oils evidences that those acyl groups, which are in greater concentrations in the oil, are those that more frequently occupy the sn-1 and sn-3 positions in the triglyceride. From this, it seems clear that the differences in the lipolysis extent of these oils, under same digestive conditions, depend mainly on the different tendency of each acyl group to hydrolyze and on its concentration in the oil, because the frequency of their presence in sn-1 and sn-3 positions of the backbone of the triglyceride is a function of the concentration of each acyl group in the oil.

##### Quantitative Relationships between Lipolysis Extent Reached and Concentrations of the Different Kinds of Acyl Groups in the Oil

In order to go into this matter in more depth, potential quantitative relationships between lipolysis yield, expressed by the in vitro bioaccessibility of the oil main components B_OMC_ in the corresponding digestates, and concentration of oil main components in the oils submitted to in vitro digestion, expressed by the molar percentages of the different kinds of acyl groups, were tested. Both kinds of data are given in Table 1 and Table 2 respectively. As mentioned, data of corn and virgin flaxseed oils were taken from previous studies [13,14]. Regarding data in Table 2 of the molar percentages the different kinds of acyl groups in each oil, it should be mentioned that that although there are four compositional data (%S, %O, %L, %Ln), only three are independent variables. This is corroborated by the correlation matrix of the molar percentages of the different kinds of acyl groups in the four oils showed in Table S6. This table shows that molar percentages of linolenic (%Ln) and of saturated (%S) acyl groups are closely related in an inverse way (R = −0.9986), which indicates that they provide similar information. The molar percentage of the other two acyl groups (%L) and (%O) are not as closely related with any other. 

In a first approach simple linear relationship between in vitro bioaccessibility of oil main components B_OMC_ in the digestates and molar percentage of the different kinds of acyl groups in the oil were tested. The results evidenced that there is a close relationship with molar percentage of saturated (%S) acyl groups and also, as expected, with the molar percentage of linolenic (%Ln) acyl groups in the oil. The equations that describe these relations are B_OMC_ = 0.226 + 0.031 (%S) with a correlation coefficient R= 0.9148 and B_OMC_ = 0.729 − 0.004 (%Ln) with a correlation coefficient R = 0.9265. The relationship of B_OCM_ with the molar percentage of oleic (%O) acyl groups is also close (B_OMC_ = 0.489 + 0.004 (%O), R = 0.9208). However, a very slight, almost non-existent relationship between in vitro bioaccessibility and molar percentage of linoleic (%L) groups was observed. 

The above equations can lead to some considerations being made. The first is that the molar percentage of saturated, linolenic and of oleic acyl groups have an important influence on the lipolysis extent reached during in vitro digestion of these oils. Secondly that the molar percentages of saturated and also of oleic acyl groups are related to B_OMC_, or to lipolysis extent, in a direct way, which is to say that the higher the concentration of saturated and of oleic acyl groups the higher B_OMC_. However, the opposite is true for the molar percentage of linolenic acyl groups: the higher the molar percentage of linolenic acyl groups the smaller the lipolysis extent and the lower the B_OMC_ values. This result shows the important negative influence of a high concentration of linolenic groups, or of a high unsaturation degree in the oil, on its lipolytic process during its in vitro digestion, in agreement with some previous studies [8,26,27]. Furthermore, these results reaffirm previous findings on the direct relationship between concentration of saturated acyl groups and lipolysis extent [28,29]. Finally, they evidence that the concentration of oleic acyl groups is also positively related with lipolysis extent, which has been proved, for the first time, in this study.

In order to deepen the study of the influence of the oil composition on the lipolysis extent reached during its in vitro digestion, equations involving two variables were tested in an attempt to find relationships, closer than the above, between in vitro bioaccessibility and concentrations of the different acyl groups in the oil. The equations obtained are indicated in Table 3. It can be observed that in the five equations shown there is a very close relationship between in vitro bioaccessibility and the molar percentage of two kinds of acyl groups. These equations demonstrate once again the direct relationships between lipolysis extent and concentration of saturated and oleic acyl groups, the weight of the molar percentage of saturated acyl groups being around ten times higher than that of the oleic groups. Likewise, it can again be observed that lipolysis extent is inversely related with the molar percentage of linolenic and linoleic acyl groups, the weight of the first being double than that of the second. Furthermore, equations involving saturated and linoleic acyl groups (or linolenic and oleic acyl groups) also have high correlation coefficients, as can be expected due to the very close relationship between %S and %Ln. In these two latter equations it is again shown that the greater the concentration of saturated or of oleic acyl groups the higher the in vitro bioaccessibility, and the opposite is true for linolenic and linoleic groups. These results suggest that the trend of saturated groups to be hydrolyzed is high, and this decreases progressively as the unsaturation degree of the acyl group increases, reaching the least tendency in linolenic groups, whereas oleic and linoleic groups show an intermediate tendency. Finally, the equation that involves these latter acyl groups also has a very high correlation coefficient, the weight of the molar percentage of oleic group being double that of the linoleic group.

Finally, the predictive values of equations given in Table 3 were analyzed with data coming from a previous study concerning virgin and refined soybean oils [11]. The introduction of the molar percentages of the different kinds of acyl groups of these oils (virgin soybean oil: %Ln = 5.5, %L = 44.7, %O = 32.5, %S = 17.3, B_OMC_ = 0.65; refined soybean oil: %Ln = 4.9, %L = 47.6, %O = 32.1, %S = 16.3, B_OMC_ = 0.66) in the different equations given in Table 3 allows one to evaluate their predictive value. It has been proved that Equations (1), (3) and (4) of Table 3 can predict the in vitro bioaccessibility of these soybean oils with a high level of approximation, which evidences their soundness. This could be carried out over a greater number of edible oils, all of them submitted to the same in vitro digestive conditions, to obtain much more general equations, which can be of interest to design mixtures of edible oils with a specific bioaccessibility in order to prepare diets for special needs with different nutritional purposes. 

#### 3.1.3. Effect of the Enrichment of Olive Oil with Different Phenolic Compounds on Lipolysis Extent and Oil Main Component Bioaccessibility Reached During In Vitro Digestion 

It has been reported that certain polyphenolic compounds, polymeric or not, are able to inhibit lipase activity and reduce the lipolysis extent reached during lipid digestion [5,38]. However, this ability has not been observed in the phenolic compounds involved in this study [14]. Table 1 shows the molar percentages of the different glyceryl species found in the digestates of olive oil enriched with various concentrations of dodecyl gallate (DG), hydroxytyrosol acetate (HTA) and *gamma*-tocopherol (γT). As this table shows, no significant differences have been found either in the molar percentage of any of the glyceryl species or in the bioaccessibility of oil main components between the digestates of the not enriched olive oils and those of the olive oils enriched with phenolic compounds. This reinforces previous results that showed no inhibitory capacity of these phenolic compounds on pancreatic lipase [14].

### 3.2. Occurrence of Oxidation Reactions During In Vitro Digestion of Olive Oil, Comparison with that of Corn and Virgin Flaxseed Oil, and Effect of Olive Oil Enrichment with Phenolic Compounds on This Issue 

In previous studies on the in vitro digestion of edible oils of different unsaturation degree it was proved that lipid oxidation takes place during this process [9,10,11,12,13,14]. This is a subject of great importance due to the toxicity of some of the oxidation compounds that can be formed, because they can be absorbed through the intestinal wall. The occurrence of lipid oxidation reactions during digestion can be evaluated either by the degradation of fatty acids and acyl groups or by the formation of oxidation compounds. 

Due to esters having greater oxidative stability than fatty acids, it may be supposed that the fatty acids released in the lipolytic process will be the candidates for oxidization if it takes place. Both, acyl groups and fatty acids can be estimated jointly by ^1^H NMR spectroscopy as indicated in the experimental section and in previous studies [13,14]. The differences between the concentration of acyl groups plus fatty acids in the oil and in the corresponding digestate will inform about the degradation of some of them during digestion due to oxidation [9,10,11,12,13,14]. 

Likewise, the formation of oxidation compounds during digestion can be evaluated by both ^1^H NMR spectroscopy and by solid phase microextraction followed by gas chromatography-mass spectrometry (SPME-GC/MS) as indicated in the experimental section [18,24]. The first technique permits one, whenever the concentration of oxidation compounds in the extract of the digestate is high enough, to detect and quantify oxidation compounds contained in it [9,10,11,12,13,14]. The second technique allows measurement of the abundance of secondary oxidation compounds volatile present in the headspace of the digestate, as indicated in the experimental section and in previous studies [9,10,11,12,13,14].

#### 3.2.1. Analysis of Potential Changes in the Concentration of Unsaturated Fatty Acids-Acyl Groups during In Vitro Digestion 

##### In Olive Oils

The molar percentages of the different kinds of fatty acids and acyl groups determined jointly in the olive oils subject of study and in their corresponding digestates are given in Table 2. It can be observed that no significant differences have been found between the molar percentages of the different kinds of acyl groups and fatty acids in both olive oils and their digestates. The lack of differences indicates that, if oxidation has taken place, this has not produced variations in the concentration of the different kinds of acyl groups+fatty acids, at a level detectable by means of ^1^H NMR spectroscopy.

##### Comparison between Changes Occurred in Olive Oil with Those of Corn and Virgin Flaxseed Oils Submitted to the Same Digestive Conditions

Table 2 also gives the same data of corn and virgin flaxseed oils and of their digestates, obtained after submission of these oils to in vitro digestion under the same conditions as in this study. These data have been taken from previous studies [13,14]. The comparison of the molar percentages of the main unsaturated acyl groups and fatty acids in these two oils and in their corresponding digestates evidence that, in both oils, oxidation took place during their in vitro digestion processes [13,14]. This was evident because the molar percentages of their main unsaturated fatty acids and acyl groups, linoleic in corn oil and linolenic in virgin flaxseed oil, have smaller values in the digestates than in the oils.

Given these results, an important consideration can be made: these olive oils have greater oxidative stability than the corn and virgin flaxseed oils previously studied. If oxidation has taken place during olive oil in vitro digestion it has occurred at such a low extent that it does not provokes measurable changes by ^1^H NMR spectroscopy in their main unsaturated acyl groups and fatty acids. The higher oxidative stability of the olive oil than that of other oils, including corn and virgin flaxseed oil, has been proved before under accelerated storage conditions [39,40,41] and it is also confirmed here under in vitro digestion conditions, reinforcing the higher quality of these olive oils from this point of view.

##### Effect of Olive Oil Enrichment with Phenolic Compounds

Taking into account that no degradation of olive oil acyl groups and fatty acids has been detected by ^1^H NMR spectroscopy, during in vitro digestion, or in other words that no oxidation measurable by this technique has been observed, it is to be expected that enrichment of these oils with phenolic compounds will not cause changes in this regard. Data in Table 2 of the molar percentages of the different kinds of fatty acids and acyl groups in oils enriched in the before mentioned phenolic compounds and in their digestates evidence this fact. The lack of oxidation during in vitro digestion of these olive oils, measurable by the absence of degradation of the main unsaturated fatty acids and acyl groups, will have as its consequence greater in vitro bioaccessibility of the added phenolic compounds than in other oils in which oxidation can take place during in vitro digestion. In other words, olive oil enriched in these bioactive compounds could have more beneficial effects on health than oils in which a higher oxidation extent can take place during in vitro digestion.

#### 3.2.2. Analysis by ^1^H NMR of the Formation of Oxidation Compounds during In Vitro Digestion

In lipid oxidation processes the degradation of fatty acids and acyl groups leads to the formation of primary and of secondary oxidation compounds which can be identified and quantified by ^1^H NMR, if they are present in enough concentration as to be detected by this technique. It is well known that the first compounds formed in oxidation processes have groups containing hydroperoxy conjugated *Z,E*- or *E,E*-dienes (HPO-c(*Z,E*)-dEs or HPO-c(*E,E*)-dEs) supported on either fatty acids or on acyl groups chains. These primary oxidation compounds are intermediate compounds in the oxidation process and can evolve to form secondary oxidation compounds. The nature of these secondary oxidation compounds is very varied [39,40,41,42], among which there are aldehydes.

##### In Olive Oils

As Table 2 shows, O_1_ and O_2_ olive oils before being submitted to in vitro digestion do not contain primary oxidation compounds, however both have a small concentration of saturated aldehydes. This does not mean that they are oxidized at all. Some olive oils, as is been mentioned in the experimental section, contain a small concentration of these compounds that could have been formed in their production process by action of the lipoxygenases on the oil unsaturated acyl groups [17]. Nevertheless, as is well known, these compounds can also be formed, in absence of these enzymes, in oxidation processes under very varied conditions. The in vitro digestion of O_1_ oil does not provoke the formation either of primary or of secondary oxidation compounds measurable by ^1^H NMR spectroscopy. As Table 2 shows, DO_1_ does not contain primary oxidation compounds and only n-alkanals are detected in a concentration similar to that found in the oil before digestion. Data in Table 2 indicate that during the in vitro digestion of O_2_ very little oxidation has taken place due to the digestate DO_2_ containing a very low concentration of hydroperoxy conjugated *Z,E*-dienes (HPO-c(*Z,E*)-dEs), which were not present in the oil before digestion. DO_2_ also contains n-alkanals but in similar concentrations to those in the original oil O_2._ For this reason, their formation could not be attributed to oxidation reactions during in vitro digestion. These results are in agreement with the absence of changes in the molar percentage of the main unsaturated fatty acids and acyl groups during the digestion of these oils studied by this technique.

##### Comparison between the Oxidation Compounds Formed in Olive Oil and in Corn and Virgin Flaxseed Oils Submitted to the Same Digestive Conditions

Table 2 gives data, taken from previous studies [13,14], regarding oxidation compounds, detected by ^1^H NMR spectroscopy, in corn C and virgin flaxseed F oils and in their digestates, obtained under the same in vitro digestion conditions as in this study. It can be observed that both oils C and F are free of oxidation compounds in concentrations detectable by ^1^H NMR spectroscopy. However, their digestates contain primary (HPO-c(*Z,E*)-dEs) and also secondary oxidation compounds (n-alkanals) which proves that, during the in vitro digestion of these oils, oxidation took place. It is worth noting the important concentration of HPO-c(*Z,E*)-dEs in the digestate of corn oil DC. Likewise, it is worth highlighting the presence of HPO-c(*Z,E*)-dEs in the digestate of virgin flaxseed oil DF, although in much smaller concentration than in the digestate of corn oil DC and that of alkanals in a similar concentration as in the digestates of both olive oils. Taking into account the much higher unsaturation degree of virgin flaxseed oil than of refined corn oil it could be expected that during the in vitro digestion the lipids of the first could reach a higher oxidation degree than the lipids of the second. However, the results indicate the opposite. This higher oxidative stability shown by the virgin flaxseed oil during in vitro digestion might be due to a higher content in natural antioxidants than refined corn oil. Again, both olive oils O_1_ and O_2_ show higher oxidative stability during in vitro digestion than the other two oils before mentioned, and this fact is of great importance from the health point of view. It must be remembered that hydroperoxydes can potentially give rise to several disorders, as has been described either in in vitro or in vivo studies [43,44]. In addition, these oxidation compounds, taking into account the lower oxidative stability of fatty acids than that of acyl groups, presumably are formed on fatty acids, which are absorbable structures. Moreover, they are precursors of a great number of secondary oxidation compounds many of which are considered responsible for different degenerative diseases [45,46]. Due to the above mentioned, the behaviour of olive oil is again better than that of the other more unsaturated oils and this reinforces the quality attributes of this oil from the health point of view.

##### Effect of Olive Oil Enrichment with Phenolic Compounds on the Formation of Oxidation Compounds during In Vitro Digestion

As commented on before the main components of olive oil either do not undergo oxidation during digestion or do so at a very low degree. For this reason, the enrichment of this kind of oil with phenolic compounds, which are able to exhibit antioxidant activity will only be significant from this point of view in those olive oils able to undergo some slight oxidation. Data in Table 2 confirm the above considerations. Olive oil O_1_, which is free of hydroperoxides and only contains a basal concentration of aldehydes, when submitted to digestion does not undergo oxidation or if it does, it is so low that it is undetectable by ^1^H NMR spectroscopy. The main components of olive oil O_2_ during digestion undergo a slightly oxidation generating a small concentration of hydroperoxides, able to be detected by ^1^H NMR spectroscopy in the lipid extract of digestate DO_2_. This oxidation is not avoided with the lowest level of enrichment in *gamma*-tocopherol tested (sample O_2_γT_1_), whereas with higher levels, as in samples O_2_γT_2_ and O_2_γT_3_, this is totally avoided. These results are in line with those obtained in previous studies [13,14]. The inhibition of the formation of hydroperoxides during digestion avoids the formation of secondary oxidation compounds of which, as mentioned, some are toxic compounds [45,46]. Bearing all the above mentioned in mind, enrichment with either dodecyl gallate, hydroxytyrosol acetate or *gamma*-tocopherols could turn olive oil into what could be called a functional food, due to the great bioaccessibility of the phenolic added compounds given the almost total absence of oxidation during in vitro digestion. At this point, it should be pointed out that different biological activities have been attributed to these phenolic compounds, such as anticancer, anti-tumoral, anti-inflammatory, inhibition of platelet aggregation, and antioxidant among others [46,47,48,49,50,51,52].

#### 3.2.3. Analysis by SPME-GC/MS of the Abundance of Volatile Oxidation Markers in the Headspace of the Digestates

In order to confirm the previous results about the occurrence or not of oxidation during digestion of the olive oils and of its extent, SPME-GC/MS was used. This technique is highly sensitive and can detect volatile compounds in very low abundances. As mentioned before, all edible oils contain a basal concentration of the most known volatile oxidation markers, and their abundance increases if oxidation takes place. For this reason, this methodology is an excellent tool for the objective pursued. 

##### In the Headspace of Olive Oil Digestates

Table 4 shows the abundances of a large number of oxidation markers of the digestates DO_1_ and DO_2_ and of the mixtures constituted by the digestive juices submitted to digestion conditions and the corresponding olive oils named O_1_DJ and O_2_DJ. A comparison between the abundances of volatile oxidation markers in the headspaces of these samples provides information about the occurrence of oxidation during digestion whenever the abundance in the headspace of DO_1_ (DO_2_) sample is higher than in the headspace of O_1_DJ (O_2_DJ). As Table 4 shows, oil O_2_ has a slightly higher oxidation level than oil O_1_, as shown by the higher abundance in all oxidation markers in the headspace of the mixture O_2_DJ than in that of the mixture O_1_DJ. This subtle difference in oxidation level, which has not been detected by ^1^H NMR in the starting oils, is also reflected in the oxidation reached by these oils during digestion. It can be observed that the headspace of DO_2_ sample has higher abundances of volatile oxidation markers than that of DO_1_. In addition, these results also indicate that, during digestion of both olive oils, a very slight oxidation has taken place, as Table 4 shows. 

##### Comparison between the Oxidation Markers Abundances in the Headspace of Olive Oil Digestates and in Those of Corn and Virgin Flaxseed Oils

In previous studies in which corn and virgin flaxseed oils were submitted to in vitro digestion, the headspaces of their digestates DC and DF and of their corresponding mixtures CDJ and FDJ (these latter have the same meaning as O_1_DJ and O_2_DJ above described), were analyzed to evaluate their abundance in volatile oxidation markers [13,14]. These data [13,14] are also given in Table 4. Although, as previously shown, both abundance and nature of the volatile oxidation markers formed in the oxidation of each kind of oil are closely dependent on oil composition [22], some considerations in this regard can be made from data in Table 4. The headspace of the unoxidized corn (virgin flaxseed) oil mixed with the digestive juices submitted to digestion conditions, which can be considered the sample reference CDJ (FDJ), has a low basal content of oxidation markers in terms of number of compounds and abundance, which is somewhat higher (or of a similar order) than that of O_1_DJ, and lower than that of O_2_DJ. However, the headspace of its digestate DC (DF) is richer in oxidation compounds than that of both DO_1_ and DO_2_ digestates, showing that oxidation occurring during olive oil in vitro digestion is less than that during corn (virgin flaxseed) oil in vitro digestion. This evidences again the higher oxidative stability of olive oils than of corn and virgin flaxseed oils, under the same in vitro digestive conditions, which may be considered very relevant from the health point of view due to the toxicity of oxidation compounds. 

##### Effect of the Enrichment of Olive Oils with Phenolic Compounds on the Oxidation Marker Abundances of Their Digestates

With data from ^1^H NMR spectroscopy, as Table 2 shows, no effect of the enrichment in phenolic compounds of olive oil O_1_ on the potential oxidation of their main components produced during in vitro digestion, was observed. This was due to both the low oxidation provoked during digestion and because this technique is not sensitive enough to detect compounds in very low concentrations. However, SPME-GC/MS is highly sensitive and is able to detect differences in the headspace of the digestates coming from olive oil samples both unenriched and enriched in phenolic compounds. As Table 5 shows, the low oxidation degree provoked during O_1_ and O_2_ olive oil digestion is clearly diminished with the enrichment in phenolic compounds, the greater the higher the concentration of the phenolic compound. Furthermore, differences between the antioxidant efficiency of the three phenolic compounds added are also evidenced by the abundances of the oxidation markers in the corresponding samples. Again, and in agreement with a previous study [14], data in Table 5 show that dodecyl gallate has higher antioxidant efficiency than hydroxytyrosol acetate, and the lowest antioxidant efficiency, under these in vitro digestion conditions, is that of *gamma*-tocopherol.

### 3.3. In Vitro Bioaccessibility of Minor Compounds Involved In the Vitro Digestion of Unenriched or Enriched Olive Oil in Phenolic Compounds 

Not only is the bioaccessibility of oil main components a very important indicator from the nutritional point of view, but so is that of the oil minor components because edible oils are vehicles of vitamins and of other bioactive compounds some of which have very interesting properties from the health point of view. Likewise, it is very important to know the fate during in vitro digestion of the added polyphenols and that of compounds formed in this process. For these reasons, the in vitro bioaccessibility of different kinds of compounds before mentioned was estimated when it was possible with the techniques used in this study.

#### 3.3.1. In Vitro Bioaccessibility of Olive Oils Minor Components

Olive oils contain squalene, sterols such as cycloartenol and 24-methylencycloartenol. Both kinds of compounds can be detected and quantified by ^1^H NMR spectroscopy, in olive oils and in their digestates. Furthermore, olive oils also contain, among other minor components, a certain number of terpenes and sesquiterpenes, which can be identified, and semiquantified by SPME-GS/MS in the headspace of both olive oils and in that of their digestates. With these data, the in vitro bioaccessibility of all these compounds can be estimated. 

Using ^1^H NMR, the quantification of squalene, in both olive oils enriched or not in phenolic compounds and in their corresponding digestates, was carried out by using the area of the singlet at 1.67 ppm due to its methylenic protons in carbon atoms C-1 and C-24, indicated in Table S5. The results prove that its concentration (near 4.76 mmol/mol [AG+FA])) is the same in olive oils and in their digestates, which means that 100 % of squalene remains bioaccessible after in vitro digestion. That is to say, B = 4.76 mmol/mol [AG+FA] and B′ = 1. This is of great importance since antioxidant, cardioprotective and anti-carcinogenic activities have been attributed to this compound [53].

Likewise, the joint quantification of sterols such as cycloartenol and 24-methylencycloartenol, either free or esterified, was also carried out in both olive oils, enriched or not with phenolic compounds, and in their corresponding digestates, by means of ^1^H NMR spectral data. For this purpose, the area of the triplet centered at 0.33 ppm, due to the overlapping of the doublets of these compounds shown in Supplementary Table S5, was used. The concentration of these compounds in olive oil is near 0.30 mmol/mol [AG+FA] and their concentration remains unchanged after the in vitro digestion, being 100% bioaccessible afterwards. That is to say, B = 0.30 mmol/mol [AG+FA] and B′ = 1. These results are in line with those observed in a previous study on the in vitro digestion of virgin flaxseed oil [14]. This is an important fact, since to these compounds have been attributed with, among other beneficial biological activities, anti-cancer, anti-obesity and anti-inflammatory activities [54,55,56]. 

As mentioned, some terpenes and sesquiterpenes are also present in olive oils. The bioaccessibility of these compounds can be estimated, using SPME-GC/MS, by the ratio between their abundances in the headspaces of the digestates and of the reference samples. Analysis of the results obtained indicates that the abundances of these compounds in the headspaces of the digestates of the olive oils enriched with different concentrations of each phenolic compound have very similar values, for which reason they are given in Table 6 as average values. As Table 6 shows, the abundances of these compounds are significantly higher in the headspace of all digestates than in O_1_DJ or O_2_DJ mixtures (which are the reference samples), probably due to the matrix effect. Furthermore, no statistically significant differences were found between the abundances in the headspace of the digestates of the oil samples both unenriched and enriched in phenolic compounds, which means that the abundances of terpenes and sesquiterpenes are not affected by the several reactions that take place during in vitro digestion. For this reason, their B′ = 1. The behaviour of this kind of compound on this occasion agrees with that observed during the in vitro digestion of virgin flaxseed oil [14]. The great in vitro bioaccessibility of terpenes and of sesquiterpenes is of great importance because some health beneficial properties have been attributed to them [57].

#### 3.3.2. In Vitro Bioaccessibility of the Phenolic Added Compounds 

The concentration of dodecyl gallate and hydroxytyrosol acetate added to olive oil O_1_, can be determined, in both olive oils and in the lipid extracts of their digestates if they are present, by ^1^H NMR, by using the area of non-overlapped signals given in Supplementary Table S5. Furthermore, due to the very low extent of oxidation undergone by oil O_1_ during digestion, it should be expected that the concentration of both phenolic compounds would remain almost unaffected by the in vitro digestion. However, no signals of any of the two phenolic compounds were found in the spectra of the lipid extracts of the digestates DO_1_DG_1_, DO_1_DG_2_ and DO_1_HTA_2_ and only signals of hydroxytyrosol acetate were detected in the spectrum of the lipid extract of the digestate of the more enriched oil in this compound, that is to say in DO_1_HTA_2_. Their absence in the lipid extracts of the above mentioned digestates could not be attributed to reactions between these phenolic compounds with digestive enzymes because the lipolysis reached, in presence or in absence of these phenolic compounds, is of a similar order. Furthermore, although some reactions between phenolic compounds and aldehydes have been described, they are not very common reactions [58]. For these reasons, the absence of these two phenolic compounds in DO_1_DG_1_, DO_1_DG_2_ and DO_1_HTA_2_ digestates could be attributed to their hydrolysis because both are esters. In fact, some previous studies on hydroxytyrosol alkyl esters have described their partial hydrolysis under digestion conditions [59,60], and although, to the best of our knowledge, the hydrolysis of alkyl gallates in digestion has not been reported, this cannot be discarded. Hydrolysis of these phenolic esters yields very polar compounds that remain in the aqueous fraction of the digestates, for which reason they could not be detected in the lipid extract of the digestates, as in a previous study [14]. It only remains to add that the hydrolyzed derived compounds from these phenolic esters (gallic acid and hydroxytyrosol) are also bioactively healthy compounds. 

However, as mentioned before, hydroxytyrosol acetate was detected in the lipid extract of the digestate of the most enriched olive oil sample, DO_1_HTA_2_, probably due to its partial hydrolysis, and *gamma*-tocopherol was also detected in the lipid extracts DO_2_γT_1_, DO_2_γT_2_ and DO_2_γT_3_, due to its liposolubility. Their concentrations were estimated from the area of the ^1^H NMR signals indicated in Table S5, and with these data the in vitro bioaccessibility of these phenolic compounds was determined. As explained in the experimental section, in vitro bioaccessibility can be defined by the ratio between the concentration of the phenolic compound, PC, in the digestate [PC]_D_, given in mmoles, and the concentration of the main components also in the digestate [FA+AG]_D_, given in moles, by the equation B_PC_ = [PC]_D_/[FA+AG]_D_. This definition gives direct information about the concentration of these bioactive compounds in the digestate, and so is comparable with that of the minor and main oil components. Table 7 gives the bioaccessibilities B_PC_ thus defined for hydroxytyrosol acetate in DO_1_HTA_2_ and for *gamma*-tocopherol in DO_2_γT_1_, DO_2_γT_2_ and DO_2_γT_3._ As expected, these values are higher the higher the enrichment degree in the oils before digestion. Likewise, in vitro bioaccessibility can also be defined by the ratio between the concentration of the compound in the lipid extract of the digestate [PC]_D_ and the concentration in the oil before digestion, [PC]_O_, both in the same units, as indicated in the equation B′_PC_ = [PC]_D_/[PC]_O_. This definition gives information about the loss of the compound during in vitro digestion, or about the fraction of the phenolic compound added to the oil that remain in the digestate. B′_PC_ data of the same compounds above mentioned are given in Table 7. It is noteworthy that in all cases the loss of these phenolic compounds during in vitro digestion of olive oils is smaller than during in vitro digestion of corn and virgin flaxseed oils enriched with similar concentrations of these phenolic compounds [13,14]. These results are in agreement with the lower oxidation extent occurring during in vitro digestion of olive oil than in that of corn and virgin flaxseed oil mentioned in previous sections. The higher in vitro bioaccessibility of hydroxytyrosol acetate and of *gamma*-tocopherol in olive oil than in the other oils that have higher unsaturation degree is an important fact. It must be remembered that hydroxytyrosol acetate has been attributed beneficial health effects, such as, anti-inflammatory, antioxidant and ability to inhibit of platelet aggregation [48,61]. Likewise, many health beneficial biological activities have been attributed to *gamma*-tocopherol [46,51,52]. For all these reasons, it seems evident that olive oil is a more suitable matrix or carrier than other oils when designing functional foods enriched in liposoluble beneficial health bioactive compounds due to their greater preservation during in vitro digestion.

#### 3.3.3. In Vitro Bioaccessibility of Compounds Formed in Secondary Reactions during In Vitro Digestion and Potential Consequences

In this study, and in other previous studies [9,10,11,12,13,14], oxidation has been considered as the main secondary reaction that takes place during in vitro digestion. It has been proved in previous sections that the oxidation extent occurring during the in vitro digestion of both olive oils subject of study is much smaller than that occurring during digestion of other more unsaturated oils such as corn and virgin flaxseed oils. As Table 2 shows, either no hydroperoxydes (primary oxidation compounds) have been detected in the olive oil digestate DO_1_ or they have been detected in DO_2_ digestate, in a much smaller concentration than in the digestates of the other oils such as DC and DF. This concentration represents the bioaccessibility B of hydroperoxydes in each digestate. Furthermore, as already mentioned, these hydroperoxydes could be expected to be absorbable, with the consequent negative effects on human health, because presumably oxidation during digestion will be produced in fatty acids rather than in acyl groups, due to the lower oxidative stability of the former.

In addition, as Table 4 shows, a higher concentration of aldehydes are present in the headspace of virgin flaxseed and corn oil digestates than in that of olive oils. All these aldehydes, although in low concentration in the case of digestates of olive oil, are also bioaccessible and susceptible to reacting either with nitrogenated compounds through Maillard-type reactions or with other structures through typical aldehyde group reactions. Finally, one consideration can be made regarding the formation of aldehydes as consequence of the oxidation during digestion. As mentioned above, the higher the unsaturation of the oil the greater the formation of aldehydes during in vitro digestion, the smaller the lipolysis extent is produced. This suggests that perhaps the ability of aldehydes to react with nitrogenated compounds such as proteins, and for this reason with enzymes, could affect lipase activity negatively. The immediate consequence could be the diminution in the lipolysis extent during digestion, in those oils which tend to be oxidized more, or in other words have lower oxidative stability and a greater facility to generate aldehydes during digestion.

## 4. Conclusions

The lipolysis extent produced during the in vitro digestion of olive oil is high, yielding an important release of monoglycerides and fatty acids, and as consequence, the in vitro bioaccessibility of the olive oil main components is also high, and both are greater than of those edible oils having higher unsaturation degrees, such as corn oil and virgin flaxseed oil. For the first time very, close quantitative relationships have been found between the composition of the oil, expressed in molar percentages of the different kinds of acyl groups, and its vitro bioaccessibility. It has been proved that the in vitro bioaccessibility of the oil main components is directly related with the concentration in the oil of saturated and of oleic acyl groups, the weight of the first being almost ten times higher than that of the second. Likewise, it has been proved that in vitro bioaccessibility of the oil main components is inversely related with the concentration of the linolenic and of linoleic acyl groups in the oil, the weight of the former being double than that of the latter. Some of the obtained equations have predictive ability and are able to predict with a high level of approximation the in vitro bioaccessibility of the main components of oils not involved in the development of these equations. It has also been demonstrated that the intake of a similar amount of edible oils of different composition in acyl groups, can have very different nutritional effects, caused by, among other reasons, the different lipolysis extent reached during digestion. These differences in lipolysis extent have as their consequence differences in the bioaccessibility of the oil main components that could be absorbed through the intestinal wall. The models developed that relate in vitro bioaccessibility of oil main components and oil composition provide a very valuable tool for designing diets for very different purposes and for special needs in which high or low lipid bioaccessibility may be required. Enrichment of olive oil with different concentrations of phenolic bioactive compounds, such as dodecyl gallate, hydroxytyrosol acetate or *gamma*-tocopherol, does not modify either the lipolysis extent reached during in vitro digestion or the in vitro bioaccessibility of oil main components. It has been demonstrated that, unlike other polyphenolic compounds, these phenolic compounds do not inhibit lipase activity.

Furthermore, the oxidation extent reached during the in vitro digestion of olive oil is very small, and in some cases is undetectable by ^1^H NMR spectroscopy but only by the abundance of volatile oxidation markers analyzed by the very sensitive SPME-GC/MS technique. In all cases, the oxidation extent reached during in vitro digestion of olive oils is much smaller than that reached during the digestion of other edible oils such as corn and virgin flaxseed oil, with the consequent repercussions on health due to the toxicity of oxidation compounds. Enrichment of olive oils with phenolic compounds either reduces the oxidation extent produced during in vitro digestion to minimum values or prevents it almost totally. It has been demonstrated again that the antioxidant efficiency of these compounds is in line with the number of phenolic groups in its molecule, which means that dodecyl gallate has higher antioxidant efficiency than hydroxytyrosol acetate, and the lowest antioxidant efficiency, under these in vitro digestion conditions, is that of *gamma*-tocopherol.

It has been shown that the concentration of minor olive oil components such as squalene, some sterols, as well as terpenes and sesquiterpenes is not modified during the in vitro digestion, these being totally bioaccessible after digestion. This is of great importance due to the different beneficial bioactive capabilities attributed to most of them. The in vitro bioaccessibility of the added phenolic compounds, which are very interesting bioactive compounds, is higher in the digestate of olive oil than in those of corn and virgin flaxseed oil. This could be related to the lower oxidation extent produced during digestion of olive oil than during digestion of corn or virgin flaxseed oil. Nevertheless, estimation of the in vitro bioaccessibility of dodecyl gallate and of hydroxytyrosol acetate—the latter at the lower concentration essayed—was not possible, probably due to their hydrolysis during digestion which yields very bioactive polar compounds that remain in the aqueous fraction of the digestate. Finally, due to the very low oxidation level reached during in vitro digestion of olive oil, the bioaccessibility of the oxidation compounds formed, if any, is much smaller in the digestate of olive oil than in those of corn and virgin flaxseed oil with the consequent repercussions on health.

Among these oxidation compounds, there are aldehydes that are generated in much greater abundance in the oils having higher unsaturation degree. The great ability of aldehydes to react with nitrogenated compounds such as proteins by reactions of a Maillard type, and for this reason with enzymes including lipases, could be underlined as a potential cause of the smaller lipolysis extent produced during the in vitro digestion of the most unsaturated oils.

## Figures and Tables

**Table 1:** Lipolysis extent. Molar percentages of triglycerides (TG), diglycerides (1,2-DG and 1,3-DG), monoglycerides (1-MG and 2-MG) and glycerol (Gol) in relation to the total glyceride structures, in olive oils (O_1_ and O_2_), in corn oil (C) and in virgin flaxseed oil (F), in the digestates of these oils (DO_1_, DO_2_,, DC and DF) and in those of the samples enriched with dodecyl gallate, hydroxytyrosol acetate and *gamma*-tocopherol (DO_1_DG_1_, DO_1_DG_2_, DO_1_HTA_1_, DO_1_HTA_2_, DO_2_γT_1_, DO_2_γT_2_,and DO_2_γT_3_) whose level of enrichment in phenolic compounds is given in brackets in mmol/mol (AG+FA)_O_. Bioaccessibility of oil main components after in vitro digestion (B_OMC_), defined by the ratio (mol [FA]+[MG])_D_/mol ([FA]+[AG])_D_), where FA means fatty acid and AG acyl groups. Different letters within each column indicate statistically significant differences among the samples (*p* < 0.05). Data of corn and virgin flaxseed oils and of their digestates were taken from previous studies [13,14].

Samples	Lipolysis Extent (molar %)						Bioaccessibility (B_OMC_)
	TG (%)	1,2-DG (%)	1,3-DG (%)	2-MG (%)	1-MG (%)	Gol (%)	
***Oils***							
O_1_	98.1 ± 0.2a	1.5 ± 0.0a	-	-	-	-	
DO_1_	13.0 ± 0.6b	13.4 ± 1.7bc	1.9 ± 0.0ab	29.9 ± 2.2ab	13.7 ± 1.8a	28.1 ± 1.7abc	0.77 ± 0.02a
O_2_	98.3 ± 1.4a	1.4 ± 0.0a	-	-	-	-	
DO_2_	13.3 ± 2.0b	16.0 ± 1.0bc	2.5 ± 0.6b	33.5 ± 2.0a	8.9 ± 0.9b	25.8 ± 1.5abc	0.74 ± 0.01a
C	99.8 ± 0.2a	1.1 ± 0.1a	-	-	-	-	
DC	22.3 ± 5.9bc	14.0 ± 1.6bc	1.8 ± 1.0ab	26.6 ± 5.6ab	4.4 ± 1.1cd	30.8 ± 1.8ab	0.67 ± 0.07a
F	99.4 ± 0.0a	1.2 ± 0.0a	-	-	-	-	
DF	33.1 ± 2.7c	18.1 ± 2.1c	4.8 ± 1.0c	21.7 ± 0.5b	2.2 ± 0.8df	20.2 ± 4.3c	0.52 ± 0.05b
***Olive oil-dodecyl gallate***							
DO_1_DG_1_ (0.12)	12.9 ± 3.8b	11.6 ± 3.7b	1.8 ± 0.6ab	28.7 ± 0.2ab	12.1 ± 1.1a	32.9 ± 6.9a	0.78 ± 0.07a
DO_1_DG_2_ (1.36)	14.9 ± 1.1b	13.8 ± 1.3bc	1.4 ± 0.6ab	28.8 ± 0.6ab	13.9 ± 1.9a	27.2 ± 1.8abc	0.75 ± 0.00a
***Olive oil-hydroxytyrosol acetate***							
DO_1_HTA_1_ (0.28)	12.0 ± 1.5b	12.6 ± 0.2b	1.7 ± 0.1ab	31.2 ± 0.7a	13.0 ± 0.4a	29.5 ± 0.5ab	0.79 ± 0.01a
DO_1_HTA_2_ (2.53)	12.2 ± 0.3b	12.9 ± 0.1bc	2.0 ± 0.2b	31.4 ± 0.3a	12.0 ± 0.4a	29.4 ± 0.7ab	0.78 ± 0.01a
***Olive oil-gamma-tocopherol***							
DO_2_γT_1_ (0.11)	14.7 ± 0.4b	15.2 ± 2.2bc	1.8 ± 0.2ab	35.4 ± 0.9a	7.1 ± 1.7bc	25.8 ± 0.1abc	0.74 ± 0.02a
DO_2_γT_2_ (1.17)	15.6 ± 1.6b	15.4 ± 0.3bc	1.8 ± 0.4ab	35.9 ± 4.3a	7.8 ± 0.3b	23.6 ± 2.4bc	0.73 ± 0.02a
DO_2_γT_3_ (12.58)	15.5 ± 1.3b	16.7 ± 0.6bc	2.2 ± 0.1b	35.1 ± 4.1a	7.9 ± 0.3b	22.5 ± 1.8bc	0.72 ± 0.02a

-: not detected.

**Table 2:** Molar percentage of the main acyl groups plus fatty acids (AG + FA), in relation to the total moles of all kinds of AG and FA, in olive oils (O_1_ and O_2_), in corn oil (C) and in virgin flaxseed oil (F), in the digestates of these oils (DO_1_, DO_2_,, DC and DF) and in those of the samples enriched with dodecyl gallate, hydroxytyrosol acetate and *gamma*-tocopherol (DO_1_DG_1_, DO_1_DG_2_, DO_1_HTA_1_, DO_1_HTA_2_, DO_2_γT_1_, DO_2_γT_2_,and DO_2_γT_3_) whose level of enrichment in phenolic compounds is given in brackets in mmol/mol (AG+FA)_O_. Concentration of some oxidation compounds, expressed by mmol per mol of AG+FA in the above samples. Different letters within each column indicate statistically significant differences among the samples (*p* < 0.05). Data of corn and virgin flaxseed oils and of their digestates were taken from previous studies [13,14].

Samples	Molar (%) of Total Acyl Groups + Fatty Acids				Oxidation Compounds (mmol/mol [AG+FA])	
	Linolenic	Linoleic	Oleic	Saturated	HPO-c(*Z,E*)-dEs	n-Alkanals
***Oil***						
O_1_	0.6 ± 0.1a	8.0 ± 0.4a	75.5 ± 0.6a	15.8 ± 0.2a	-	0.12 ± 0.00a
DO_1_	0.7 ± 0.0a	7.7 ± 0.1a	75.4 ± 0.0a	16.3 ± 0.1a	-	0.08 ± 0.00a
O_2_	0.7 ± 0.1a	8.0 ± 0.1a	75.1 ± 0.6a	16.2 ± 0.5a	-	0.10 ± 0.00a
DO_2_	0.7 ± 0.0a	8.0 ± 0.1a	74.4 ± 0.4a	16.9 ± 0.6a	0.26 ± 0.04ac	0.08 ± 0.02a
C	0.6 ± 0.0a	49.2 ± 0.5b	34.1 ± 0.3b	16.1 ± 0.1a	-	-
DC	0.6 ± 0.1a	41.3 ± 0.0c	42.6 ± 0.2c	15.5 ± 0.0a	1.82 ± 0.31b	-
F	55.7 ± 0.0b	14.2 ± 0.3d	20.5 ± 1.2d	9.5 ± 0.9b	-	-
DF	47.9 ± 0.8c	14.1 ± 0.7d	25.7 ± 3.9e	12.3 ± 3.5b	0.39 ± 0.04c	0.09 ± 0.00a
***Olive oil-dodecyl gallate***					-	
DO_1_DG_1_ (0.12)	0.9 ± 0.2a	8.0 ± 0.9a	74.8 ± 0.6a	16.3 ± 0.0a	-	0.11 ± 0.03a
DO_1_DG_2_ (1.36)	0.7 ± 0.1a	8.2 ± 0.1a	75.2 ± 0.2a	15.9 ± 0.2a	-	0.10 ± 0.04a
***Olive oil-hydroxytyrosol acetate***					-	
DO_1_HTA_1_ (0.28)	0.8 ± 0.1a	7.4 ± 0.4a	75.2 ± 0.2a	16.6 ± 0.2a	-	0.08 ± 0.02a
DO_1_HTA_2_ (2.53)	0.8 ± 0.1a	7.9 ± 0.7a	75.1 ± 0.4a	16.3 ± 0.3a	-	0.08 ± 0.02a
***Olive oil-gamma-tocopherol***						
DO_2_γT_1_ (0.11)	0.7 ± 0.0a	7.8 ± 0.2a	74.2 ± 0.2a	17.4 ± 0.0a	0.27 ± 0.01ac	0.08 ± 0.00a
DO_2_γT_2_ (1.17)	0.6 ± 0.1a	7.6 ± 0.1a	74.1 ± 0.2a	17.7 ± 0.0a	-	0.07 ± 0.00a
DO_2_γT_3_ (12.58)	0.8 ± 0.1a	7.8 ± 0.1a	74.2 ± 0.2a	17.2 ± 0.2a	-	0.09 ± 0.00a

-: not detected.

**Table 3:** Coefficients of the equations B_OMC_ = a + b X_1_ + c X_2_ that relate the in vitro bioaccessibility of the oil main components (B_OMC_) and the molar percentage of certain acyl groups saturated (%S), oleic (%O), linoleic (%L) or linolenic (%Ln) in the oil before digestion, together with their correlation coefficients R.

Equation Number	Variables	Equation Coefficients		Correlation Coefficient	
	X_1_, X_2_	a	b	c	R
1	%S, %O	0.307	0.018	0.002	0.9911
2	%S, %L	0.230	0.034	−0.002	0.9872
3	%Ln, %O	0.601	−0.002	0.002	0.9944
4	%Ln, %L	0.774	−0.004	−0.002	0.9941
5	%O, %L	0.390	0.004	0.002	0.9947

**Table 4:** Abundances of some oxidation markers extracted and identified by SPME-GC/MS in the headspace of mixture of digestive juices and olive oils, corn oil and virgin flaxseed oil (O_1_DJ, O_2_DJ, CDJ and FDJ) and in the digestate of these oils (DO_1_, DO_2_, DC and DF). Data are expressed as area counts of the mass spectra base peak (Bp) of each compound multiplied by 10^−6^, and obtained as average of two determinations together with their standard deviations. Data of corn and virgin flaxseed oils were taken from previous studies [13,14].

Compound (Molecular Weight)	Bp	O_1_DJ	DO_1_	O_2_DJ	DO_2_	CDJ	DC	FDJ	DF
Aldehydes									
*Alkanals*									
Pentanal (86) *	44	11.2 ± 0.2	41.2 ± 11.8	27.0 ± 3.3	71.7 ± 4.9	17.8 ± 0.9	41.4 ± 1.8	10.6 ± 3.8	66.0 ± 5.6
Hexanal (100) *	44	3.7 ± 0.2	14.2 ± 0.1	18.9 ± 1.9	26.6 ± 0.6	12.9 ± 3.2	76.0 ± 3.1	7.9 ± 2.2	63.2 ± 9.9
Heptanal (114) *	70	0.6 ± 0.1	2.9 ± 0.3	1.7 ± 0.4	2.7 ± 0.2	0.7 ± 0.0	3.0 ± 1.2	0.5 ± 0.1	4.1 ± 0.0
Octanal (128) *	41	1.9 ± 0.4	9.9 ± 0.7	7.0 ± 2.1	8.4 ± 0.0	-	-	-	7.7 ± 0.5
Nonanal (142) *	57	2.9 ± 0.2	16.3 ± 3.1	11.9 ± 3.8	12.8 ± 1.0	3.3 ± 0.2	11.4 ± 0.3	3.0 ± 0.8	12.1 ± 1.2
***Total***		**20.3 ± 1.1**	**84.5 ± 16.0**	**66.5 ± 11.5**	**122.2 ± 6.7**	**34.7 ± 4.3**	**131.8 ± 6.4**	**19.3 ± 6.9**	**152.9 ± 17.2**
*(E)-2-Alkenals*									
(*E*)-2-Butenal (70) *	70	-	-	-	-	19.5 ± 2.8	20.6 ± 3.4	-	-
(*E*)-2-Butenal-2-methyl (84)	55	1.2 ± 0.0	2.0 ± 0.3	1.4 ± 0.2	1.9 ± 0.2	-	-	-	-
(*E*)-2-Pentenal (84)	41	-	-	-	-	-	-	2.2 ± 0.3	10.7 ± 0.3
(*E*)-2-Hexenal (98) *	41	0.7 ± 0.1	2.2 ± 0.3	2.2 ± 0.1	3.4 ± 0.2	-	-	0.4 ± 0.1	1.4 ± 0.0
(*Z*)-4-Heptenal (112)	41	-	-	-	-	-	-	1.5 ± 0.1	6.1 ± 1.4
(*E*)-2-Heptenal (112)	41	0.8 ± 0.1	6.8 ± 0.9	2.3 ± 0.0	9.4 ± 2.1	2.3 ± 0.3	47.4 ± 8.1	-	-
(*E*)-2-Nonenal (140) *	55	0.4 ± 0.0	1.4 ± 0.1	0.5 ± 0.0	1.9 ± 0.4	-	1.2 ± 0.2	-	0.3 ± 0.0
***Total***		**3.1 ± 0.2**	**12.4 ± 1.6**	**6.4 ± 0.3**	**16.6 ± 2.9**	**21.8 ± 3.1**	**69.2 ± 11.7**	**4.1 ± 0.5**	**18.5 ± 1.7**
*2,4-Alkadienals*									
(*E,E*)-2,4-Hexadienal (96) *	81	0.5 ± 0.0	1.6 ± 0.4	3.1 ± 0.4	8.3 ± 0.7	-	-	-	2.9 ± 0.2
(*Z,E*)-2,4-Heptadienal (110)	81	0.6 ± 0.2	2.6 ± 0.1	0.3 ± 0.0	2.3 ± 0.3	-	-	3.3 ± 0.9	19.7 ± 0.6
(*E,E*)-2,4-Heptadienal (110) *	81	-	1.3 ± 0.1	-	1.0 ± 0.0	-	-	2.2 ± 0.4	21.3 ± 0.7
(*E,E*)-2,4-Nonadienal (138)	81	-	-	-	-	-	1.0 ± 0.1	-	-
(*Z,E*)-2,4-Decadienal (152)	81	-	-	-	-	-	0.6 ± 0.0	-	-
(*E,E*)-2,4-Decadienal (152) *	81	-	-	-	-	-	0.8 ± 0.1	-	-
***Total***		**1.1 ± 0.2**	**5.5 ± 0.6**	**3.4 ± 0.4**	**11.6 ± 1.0**	**-**	**2.4 ± 0.2**	**5.5 ± 1.3**	**43.9 ± 1.5**
**Furan derivatives**									
Furan, 2-ethyl (96) *	81	-	-	0.2 ± 0.0	0.6 ± 0.0	-	-	1.9±0.0	6.3±2.6
Furan, 2-butyl (124)	81	-	-	-	-	-	0.5±0.0	0.3±0.0	0.5±0.1
Furan, 2-pentyl (138) *	81	1.7 ± 0.4	14.5 ± 0.9	2.8 ± 0.8	13.2 ± 0.2	4.7 ± 1.4	21.5 ± 1.5	4.2 ± 1.1	11.7 ± 0.7
***Total***		**1.7 ± 0.4**	**14.5 ± 0.9**	**3.0 ± 0.8**	**13.8 ± 0.2**	**4.7 ± 1.4**	**22.0 ± 1.5**	**6.4 ± 1.1**	**18.5 ± 3.4**

* Asterisked compounds were acquired commercially and used as standards for identification purposes; -: not detected.

**Table 5:** Abundances of some oxidation markers extracted and identified by SPME-GC/MS in the headspace of digestates of non-enriched olive oils (DO_1_ and DO_2_) and in those of the samples enriched with dodecyl gallate, hydroxytyrosol acetate and *gamma*-tocopherol (DO_1_DG_1_, DO_1_DG_2_, DO_1_HTA_1_, DO_1_HTA_2_, DO_2_γT_1_, DO_2_γT_2_ and DO_2_γT_3_) whose level of enrichment in phenolic compounds is given in brackets in mmol/mol (AG+FA)_O_. Data are expressed as area counts of the mass spectra base peak (Bp) of each compound multiplied by 10^-6^, and obtained as average of two determinations together with their standard deviations.

Compound (Molecular Weight)	Bp	DO_1_	DO_1_DG_1_ (0.12)	DO_1_DG_2_ (1.36)	DO_1_HTA_1_ (0.28)	DO_1_HTA_2_ (2.53)	DO_2_	DO_2_γT_1_ (0.11)	DO_2_γT_2_ (1.17)	DO_2_γT_3_ (12.58)
Aldehydes										
*Alkanals*										
Pentanal (86) *	44	41.2 ± 11.8	14.9 ± 4.0	15.6 ± 0.1	32.7 ± 5.0	22.2 ± 1.9	71.7 ± 4.9	73.1 ± 15.0	50.0 ± 0.2	34.7 ± 0.6
Hexanal (100) *	44	14.2 ± 0.1	5.7 ± 0.3	5.6 ± 1.5	11.3 ± 1.3	8.4 ± 0.3	26.6 ± 0.6	23.0 ± 1.1	19.6 ± 1.0	16.8 ± 0.2
Heptanal (114) *	70	2.9 ± 0.3	1.1 ± 0.2	1.1 ± 0.0	1.0 ± 0.0	1.7 ± 0.3	2.7 ± 0.2	2.6 ± 0.2	2.0 ± 0.2	1.5 ± 0.2
Octanal (128) *	41	9.9 ± 0.7	5.2 ± 0.1	3.7 ± 0.1	3.7 ± 0.1	6.1 ± 0.6	8.4 ± 0.0	10.4 ± 0.5	8.0 ± 0.3	-
Nonanal (142) *	57	16.3 ± 3.1	6.1 ± 0.5	6.3 ± 0.3	6.0 ± 0.4	10.2 ± 1.6	12.8 ± 1.0	11.3 ± 1.0	9.2 ± 0.4	7.9 ± 0.0
***Total***		**84.5 ± 16.0**	**33.0 ± 5.1**	**32.3 ± 2.0**	**54.7 ± 6.8**	**48.6 ± 4.7**	**122.2 ± 6.7**	**120.4 ± 17.8**	**88.8 ± 2.1**	**60.9 ± 1.0**
*(E)-2-Alkenals*										
(*E*)-2-Butenal-2-methyl (84)	55	2.0 ± 0.3	1.3 ± 0.3	2.4 ± 0.3	2.1 ± 0.2	1.9 ± 0.5	1.9 ± 0.2	2.4 ± 0.4	2.0 ± 0.2	1.3 ± 0.5
(*E*)-2-Hexenal (98) *	41	2.2 ± 0.3	0.9 ± 0.2	0.8 ± 0.2	1.5 ± 0.2	0.8 ± 0.1	3.4 ± 0.2	3.2 ± 0.1	2.6 ± 0.2	2.1 ± 0.4
(*E*)-2-Heptenal (112)	41	6.8 ± 0.9	2.3 ± 0.2	2.2 ± 0.1	2.2 ± 0.2	2.7 ± 0.6	9.4 ± 2.1	8.8 ± 0.1	5.8 ± 0.4	3.8 ± 0.4
(*E*)-2-Nonenal (140) *	55	1.4 ± 0.1	0.6 ± 0.1	0.8 ± 0.1	1.0 ± 0.1	0.9 ± 0.0	1.9 ± 0.4	2.0 ± 0.2	1.0 ± 0.0	1.0 ± 0.0
***Total***		**12.4 ± 1.6**	**5.1 ± 0.8**	**6.2 ± 0.7**	**6.8 ± 0.7**	**6.3 ± 1.2**	**16.6 ± 2.9**	**16.4 ± 0.8**	**11.4 ± 0.8**	**8.2 ± 1.3**
*2,4-Alkadienals*										
(*E,E*)-2,4-Hexadienal (96) *	81	1.6 ± 0.4	1.1 ± 0.2	0.6 ± 0.2	1.1 ± 0.2	0.4 ± 0.1	8.3 ± 0.7	8.9 ± 0.1	7.0 ± 1.8	5.4 ± 0.7
(*Z,E*)-2,4-Heptadienal (110)	81	2.6 ± 0.1	1.3 ± 0.3	0.9 ± 0.0	2.0 ± 0.2	1.5 ± 0.4	2.3 ± 0.3	2.4 ± 0.1	1.8 ± 0.3	1.0 ± 0.0
(*E,E*)-2,4-Heptadienal (110) *	81	1.3 ± 0.1	0.5 ± 0.1	-	0.5 ± 0.0	0.4 ± 0.0	1.0 ± 0.0	1.2 ± 0.1	0.9 ± 0.2	0.6 ± 0.2
***Total***		**5.5 ± 0.6**	**2.9 ± 0.6**	**1.5 ± 0.2**	**3.6 ± 0.4**	**2.3 ± 0.5**	**11.6 ± 1.0**	**12.5 ± 0.3**	**9.7 ± 2.3**	**7.0 ± 0.9**
**Furan derivatives**										
Furan, 2-ethyl (96) *	81	-	-	-	-	-	0.6 ± 0.0	0.5 ± 0.1	0.2 ± 0.1	-
Furan, 2-pentyl (138) *	81	14.5 ± 0.9	5.4 ± 1.3	5.3 ± 0.5	8.7 ± 1.2	6.1 ± 0.1	13.2 ± 0.2	11.9 ± 1.0	11.4 ± 1.2	11.2 ± 0.7
***Total***		**14.5 ± 0.9**	**5.4 ± 1.3**	**5.3 ± 0.5**	**8.7 ± 1.2**	**6.1 ± 0.1**	**13.8 ± 0.2**	**12.4 ± 1.1**	**11.6 ± 1.3**	**11.2 ± 0.7**

* Asterisked compounds were acquired commercially and used as standards for identification purposes; -: not detected.

**Table 6:** Terpenes and sesquiterpenes of olive oil, detected by SPME-GC/MS in the headspaces of the mixture of digestive juices submitted to digestive conditions and olive oils (O_1_DJ and O_2_DJ), of the digestate of these oils (DO_1_ and DO_2_) and of the samples enriched with different levels of dodecyl gallate, hydroxytyrosol acetate, and *gamma*-tocopherol (DO_1_DG, DO_1_HTA, DO_2_γT). Data are average abundances expressed as area counts of the mass spectra base peak (Bp) of each compound multiplied by 10^-6^, together with their standard deviations. For samples enriched with phenolic compounds data given are average values of the abundances of the headspace of digestates coming from samples having different enrichment levels of phenolic compounds. Different letters within each row of each oil indicate statistically significant differences among the samples (*p* < 0.05).

*Terpenes and Sesquiterpenes*	Bp	O_1_DJ	DO_1_	DO_1_DG_average_	DO_1_HTA_average_	O_2_DJ	DO_2_	DO_2_γT_average_
*alpha*-pinene *	93	0.1 ± 0.0a	0.2 ± 0.1b	0.2 ± 0.0b	0.2 ± 0.0b	0.1 ± 0.0a	0.3 ± 0.0b	0.3 ± 0.0b
Limonene *	68	0.4 ± 0.0a	2.0 ± 0.3b	2.2 ± 0.3b	2.1 ± 0.2b	0.6 ± 0.0a	3.5 ± 0.2b	3.5 ± 0.4b
*beta*-ocimene *	93	0.2 ± 0.0a	0.3 ± 0.0b	0.3 ± 0.0b	0.4 ± 0.1b	0.3 ± 0.1a	0.6 ± 0.1b	0.6 ± 0.1b
*alpha*-gurjunene	93	0.1 ± 0.0a	0.2 ± 0.0b	0.2 ± 0.0b	0.2 ± 0.0b	0.1 ± 0.0a	0.2 ± 0.0b	0.2 ± 0.0b
*alpha*-copaene	119	0.1 ± 0.0a	0.4 ± 0.0b	0.4 ± 0.0b	0.4 ± 0.0b	0.2 ± 0.0a	0.5 ± 0.0b	0.5 ± 0.0b
*alpha*-guaiene	93	0.1±0.0a	0.2 ± 0.0b	0.2 ± 0.0b	0.3 ± 0.0b	0.1 ± 0.0a	0.3 ± 0.0b	0.3 ± 0.0b
*alpha*-farnesane	161	0.2 ± 0.0a	0.5 ± 0.0b	0.5 ± 0.1b	0.5 ± 0.1b	0.2 ± 0.0a	0.6 ± 0.0b	0.5 ± 0.0b

* Asterisked compounds were acquired commercially and used as standards for identification purposes.

**Table 7:** Bioaccessibility of hydroxytyrosol acetate (HTA) and *gamma*-tocopherol (γT) in the digestates of the different samples in which these compounds are present, expressed in two different ways. B = (mmol PC_D_/mol (AF+GA)_D_) and B′ = (mmol PC_D_/mmol PC_O_). Values are the average of two determinations together with their standard deviations.

Samples	B_HTA_	B′_HTA_	B_γT_	B′_γT_
DO_1_HTA_2_	1.63 ± 0.03	0.64 ± 0.01	n.a.	n.a.
DO_2_γT_1_	n.a.	n.a.	0.07 ± 0.00	0.63 ± 0.01
DO_2_γT_2_	n.a.	n.a.	0.85 ± 0.02	0.72 ± 0.02
DO_2_γT_3_	n.a.	n.a.	10.57 ± 0.11	0.84 ± 0.01

n.a.: not applicable.

## Supplementary Materials

The following are available online at https://www.mdpi.com/2076-3921/9/6/543/s1, Table S1: Composition and pH values of the juices employed in the in vitro digestion model employed in this study; Table S2: Chemical shift assignments and multiplicities of the ^1^H NMR signals in CDCl_3_ of protons of glycerides; Table S3: Chemical shift assignments and multiplicities of the ^1^H NMR signals in CDCl_3_ of protons of acyl groups and fatty acids; Table S4: Chemical shift assignments and multiplicities of the ^1^H NMR signals in CDCl_3_ of protons of some oxidation compounds detected in the digestates and formed during the in vitro digestion; Table S5: Chemical shift assignments and multiplicities of the ^1^H NMR signals in CDCl_3_ of protons of cycloartenol and methylencycloartenol, esters of cycloartenol and methylencycloartenol, squalene, *gamma*-tocopherols, hydroxytyrosol acetate and dodecyl gallate detected in the samples before and after in vitro digestion; Table S6: Correlation matrix between the molar percentages of the different kinds of acyl groups present in the four oils involved in this study; Operating conditions for the acquisition of the ^1^H NMR Spectra; Equations used for the quantification from ^1^H NMR spectral data of several compounds present in the starting samples and/or in the lipid extract of the digestates; Operating conditions for the SPME-GC/MS Experiments.
