# Supplementary material for: Study of the In Vitro Digestion of Olive Oil Enriched or Not with Antioxidant Phenolic Compounds. Relationships between Bioaccessibility of Main Components of Different Oils and Their Composition
Source: Antioxidants (Basel). 2020 Jun 20;9(6):543. doi: 10.3390/antiox9060543 (PMC7346224; doi:10.3390/antiox9060543)
Supplement: Supplementary file 1 [file antioxidants-09-00543-s001.pdf]

SUPPLEMENTARY MATERIAL

Study of the In Vitro Digestion of Olive Oil Enriched or not with Antioxidant Phenolic Compounds. Relationships Between Bioaccessibility Of Main Components of Different Oils and Their Composition.

J. Alberdi-Cedeño, María L. Ibargoitia and María D. Guillén*

Food Technology. Faculty of Pharmacy. Lascaray Research Center. University of the Basque Country (UPV-EHU). Paseo de la Universidad nº 7, 01006 Vitoria-Gasteiz, Spain, Telf: 34-945-013081, Fax: 34-945-013014. E-mail: mariadolores.guillen@ehu.es*

Table S1. Composition and pH values of the juices employed in the *in vitro* digestion model employed in this study.

Components	Saliva	Gastric juice	Duodenal juice	Bile juice
KCl (mmol/L)	12.02	11.06	7.57	5.05
NaCl (mmol/L)	5.10	47.09	119.98	89.99
$NaHCO_3$ (mmol/L)	20.17	-	40.33	68.86
NaH_2PO_4 (mmol/L)	7.40	0.22	-	-
NH_4Cl (mmol/L)	-	5.72	-	-
KH_2PO_4 (mmol/L)	-	-	0.59	-
Na_2SO_4 (mmol/L)	4.79	-	-	-
KSCN (mmol/L)	2.06	-	-	-
$MgCl_2$ (mmol/L)	-	-	0.53	-
$CaCl_2$*$2H_2O$ (mmol/L)	-	2.72	1.36	1.51
HCl (37%) (mL/L)	-	6.50	0.18	0.15
Urea (mmol/L)	3.33	1.42	1.67	4.16
Glucose (mmol/L)	-	3.61	-	-
Glucuronic acid (mmol/L)	-	0.10	-	-
Uric acid (mmol/L)	0.09	-	-	-
Glucoseamine hydrochloride (mmol/L)	-	1.53	-	-
Bovine serum albumin (g/L)	-	1.00	1.00	1.80
Mucin (g/L)	0.025	3.00	-	-
A. oryzae α-amylase (g/L)	0.29	-	-	-
A. niger lipase (U/mL)	-	100	-	-
Pepsin (g/L)	-	2.50	-	-
Pancreatin (g/L)	-	-	9.00	-
Lipase type II from porcine pancreas (g/L)	-	-	1.50	-
Bovine bile extract (g/L)	-	-	-	18.75
**pH**	6.9 ± 0.0	1.4 ± 0.1	8.1 ± 0.0	8.2 ± 0.1

Table S2. Chemical shift assignments and multiplicities of the ^{1}H NMR signals in $CDCl_3$ of protons of glycerides. TG: triglycerides; DG: diglycerides; MG: monoglycerides.

Signal	Chemical shift (ppm)	Multiplicity	Type of protons	Structures
			**Glycerides structure protons**	
**I**	3.65	ddd	ROCH$_2$–CHOH–C**H$_2$**OH	glyceryl group in **1-MG**
**J**	3.73	m*	ROCH$_2$–CH(O*R*')–C**H$_2$**OH	glyceryl group in **1,2-DG**
**K**	3.84	m*	HOC**H$_2$**–CH(O*R*)–C**H$_2$**OH	glyceryl group in **2-MG**
**L**	3.94	m	ROCH$_2$–C**H**OH–CH$_2$OH	glyceryl group in **1-MG**
**M**	4.05–4.21	m	ROC**H$_2$**–C**H**OH–C**H$_2$**OR'	glyceryl group in **1,3-DG**
**N**	4.18	ddd	ROC**H$_2$**–CHOH–CH$_2$OH	glyceryl group in **1-MG**
**O**	4.22	dd,dd	ROC**H$_2$**–CH(O*R*')–C**H$_2$**OR''	glyceryl group in **TG**
**P**	4.28	ddd	ROC**H$_2$**–CH(O*R*')–CH$_2$OH	glyceryl group in **1,2-DG**
**Q**	4.93	m	HOCH$_2$–C**H**(O*R*)–CH$_2$OH	glyceryl group in **2-MG**
**R**	5.08	m	ROCH$_2$–C**H**(O*R*')–CH$_2$OH	glyceryl group in **1,2-DG**

Abbreviations: d: doublet; m: multiplet.

*This signal shows different multiplicity if the spectrum, is acquired from the pure compound or taking part in the mixture.

**The intensity of some of these signals, together with signal F of Table S3, were used to estimate the molar percentages of different kinds of glyceryl structures using the equations [eq. S1-eq. S10].

***The assignment of the ^{1}H NMR signals of the protons was made as in previous studies (Guillén & Uriarte, 2012a; Nieva-Echevarría et al., 2014).

Table S3. Chemical shift assignments and multiplicities of the ^1H NMR signals in $CDCl_3$ of protons of acyl groups and fatty acids. AG: acyl groups; FA: fatty acids.

Signal	Chemical shift (ppm)	Multiplicity	Type of protons	Structures
				**Main acyl groups (AG) and fatty acids (FA)**
**A**	0.88	t	–C$\underline{\mathbf{H}}_3$	saturated and monounsaturated **ω-9** in **AG** and **FA**
	0.89	t	–C$\underline{\mathbf{H}}_3$	linoleic in **AG** and **FA**
**B**	0.97	t	–C$\underline{\mathbf{H}}_3$	linolenic in **AG** and **FA**
**C**	1.19–1.42	m**	–(C$\underline{\mathbf{H}}_2$)$_n$–	**AG** and **FA**
	1.61	m	–OCO–CH$_2$–C$\underline{\mathbf{H}}_2$–	**AG** in **TG**
	1.62	m	–OCO–CH$_2$–C$\underline{\mathbf{H}}_2$–	**AG** in **1,2-DG**
**D**	1.63	m	–OCO–CH$_2$–C$\underline{\mathbf{H}}_2$–, COOH–CH$_2$–C$\underline{\mathbf{H}}_2$–	**AG** in **1,3-DG**, **1-MG** and **FA**
	1.64	m	–OCO–CH$_2$–C$\underline{\mathbf{H}}_2$–	**AG** in **2-MG**
**E***	1.92–2.15	m***	–C$\underline{\mathbf{H}}_2$–CH=CH–	**AG** and **FA**
	2.26–2.36	dt	–OCO–C$\underline{\mathbf{H}}_2$–	**AG** in **TG**
	2.33	m	–OCO–C$\underline{\mathbf{H}}_2$–	**AG** in **1,2-DG**
**F***	2.35	t	–OCO–C$\underline{\mathbf{H}}_2$–, COOH–C$\underline{\mathbf{H}}_2$–	**AG** in **1,3-DG**, **1-MG** and **FA**
	2.38	t	–OCO–C$\underline{\mathbf{H}}_2$–	**AG** in **2-MG**
**G***	2.77	t	=HC–C$\underline{\mathbf{H}}_2$–CH=	linoleic in **AG** and **FA**
**H***	2.80	t	=HC–C$\underline{\mathbf{H}}_2$–CH=	linolenic in **AG** and **FA**

Abbreviations: d: doublet; t: triplet; m: multiplet.

*The intensity of these signals was used to estimate the molar percentage of the main acyl groups plus fatty acids by using equations [eq. S11-eq. S14].

**Overlapping of multiplets of methylenic protons in the different acyl groups either in β-position, or further, in relation to double bonds, or in γ-position, or further, in relation to the carbonyl group.

***Overlapping of multiplets of the α-methylenic protons in relation to a single double bond of the different unsaturated acyl groups.

****The assignment of the ^1H NMR signals of the protons was made as in previous studies (Guillén & Ruiz, 2003; Nieva-Echevarría et al., 2014).

Table S4. Chemical shift assignments and multiplicities of the ^1H NMR signals in $CDCl_3$ of protons of some oxidation compounds detected in the digestates and formed during the *in vitro* digestion.

Signal	Chemical shift (ppm)	Multiplicity	Type of protons	Structures
**Oxidation Compounds (OC)**				
**Conjugated dienic systems associated with hydroperoxy groups**				
-	5.51	dtm	–C**H**=CH–CH=CH–	(*Z,E*)-conjugated double bonds associated with hydroperoxy group (OOH) in octadecadienoic **AG** and **FA** **HPO-c(*Z,E*)-dEs**
-	5.56	ddm		
-	6.00	ddtd		
**b**	***6.58***	dddd		
**Aldehydes**				
**f**	***9.75***	t	-C**H**O	**n-alkanals**
	2.40	dt	$-CH_2-$	

Abbreviations: d: doublet; t: triplet; m: multiplet.

*The intensities of the signals indicated in bold, together with signal D of Table S3, were used to estimate the concentration (mmol/molAG+FA) using the equation [eq. S15].

**The assignment of the ^1H NMR signals of the protons was made with the aid of standard compounds and with the data taken from literature (Guillén & Ruiz, 2005).

Table S5. Chemical shift assignments and multiplicities of the ^1H NMR signals in $CDCl_3$ of protons of cycloartenol and methylencycloartenol, esters of cycloartenol and methylencycloartenol, *gamma*-tocopherols, hydroxytyrosol acetate and dodecyl gallate detected in the samples before and after *in vitro* digestion.

Signal	Chemical shift (ppm)	Multiplicity	Type of protons	Compounds
**Sterols**				
**4,4´-DiMe-St.**	***0.33*****	d	-C**H₂**- (exo, C-19)	Cycloartenol/ 24-Methylenecycloartenol
**4,4´-DiMe-St´.**	***0.34*****	d	C**H₂**- (exo, C-19)	Esters of Cycloartenol/ 24-Methylenecycloartenol
**Squalene**				
**SQ**	***1.67*****	s	-C**H₃** (C-1; C-24)	Squalene
***gamma*-Tocopherol**				
**γT**	***6.36*****	s	-C**H** (C-5)	*gamma*-tocopherol
**Hydroxytyrosol acetate**				
**HTA**	6.60	dd	Ar**H** (C-8)	Hydroxytyrosol acetate
	6.75**	d	Ar**H** (C-4)	
	6.78	d	Ar**H** (C-7)	
**Dodecyl gallate**				
**DG**	***7.20*****	s	Ar**H** (C-3; C-7)	Dodecyl gallate

Abbreviations: s: singlet; d: doublet.

*The intensity of these signals, together with signal D of Table S3, were used to estimate the concentration (mmol/molAG+FA) using the equation [eq. S15].

**Assignment was made with the aid of standard compounds and with the data taken from the literature (Baker & Mayers, 1991; Pogliani et al., 1994; Kubo et al., 2002; Kawai et al., 2007).

References

Baker, J.K., Myers, C.W. One-dimensional and two-dimensional ^{1}H-and ^{13}C-nuclear magnetic resonance (NMR) analysis of vitamin E raw materials or analytical reference standards. *Pharm. Res.* **1991**, *8*(6), 763-770.

Guillén, M.D.; Ruiz, A. Rapid simultaneous determination by proton NMR of unsaturation and composition of acyl groups in vegetable oils. *Eur J Lipid Sci Technol* **2003**, *105*(11), 688-696.

Guillén, M.D.; Ruiz, A. Oxidation process of oils with high content of linoleic acyl groups and formation of toxic hydroperoxy-and hydroxyalkenals. A study by ^{1}H nuclear magnetic resonance. *J. Agric. Food Chem.* **2005**, *85*(14), 2413-2420.

Guillén, M.D.; Uriarte, P.S. Study by ^{1}H NMR spectroscopy of the evolution of extra virgin olive oil composition submitted to frying temperature in an industrial fryer for a prolonged period of time. *Food Chem.* **2012a**, *134*(1), 162-172.

Kawai, S., Takada, Y., Tsuchida, S., Kado, R., Kimura, J. Sterols from bivalves Calyptogena soyoae and Bathymodiolus septemdierum living in deep sea. *Fish. Res.* **2007**, *73*(4), 902-906.

Kubo, I., Xiao, P., Nihei, K. I., Fujita, K. I., Yamagiwa, Y., Kamikawa, T. Molecular design of antifungal agents. *J. Agric. Food Chem.* **2002**, *50*(14), 3992-3998.

Nieva-Echevarría, B.; Goicoechea, E.; Manzanos, M.J.; Guillén, M.D. A method based on ^{1}H NMR spectral data useful to evaluate the hydrolysis level in complex lipid mixtures. *Food Res. Int.* **2014**, 66, 379–387.

Pogliani, L., Ceruti, M., Ricchiardi, G., Viterbo, D. An NMR and molecular mechanics study of squalene and squalene derivatives. *Chem. Phys. Lipids* **1994**, *70*(1), 21-34.

Table S6. Correlation matrix between the molar percentages of the different kinds of acyl groups, linoleic (%L), linolenic (%Ln), oleic (%O) and saturated (%S), present in the four oils involved in this study.

	**%L**	**%Ln**	**%O**	**%S**
**%L**	1.0000	0.1913	0.5363	0.2042
**%Ln**	0.1913	1.0000	0.7259	- 0.9986
**%O**	0.5363	0.7259	1.0000	0.7153
**%S**	0.2042	- 0.9986	0.7153	1.0000

Operating Conditions for the Acquisition of the ^1H NMR Spectra

The ^1H NMR spectra were acquired in duplicate using a Bruker Avance 400 spectrometer operating at 400 MHz. For this purpose, the samples (approximately 0.16 g) were dissolved in 400 μL of deuterated chloroform, which contained tetramethylsilane (TMS), as internal reference (Cortec, Paris, France). First, a standard ^1H NMR spectrum was acquired and in a second step, a NOESYGPPS experiment consisting of a one-dimensional ^1H NMR pulse sequence with selective suppression of certain strong signals was carried out. This NOESYGPPS experiment allows one to obtain a ^1H NMR spectrum with a greater sensitivity than that of the standard single pulse ^1H NMR experiment (Ruiz-Aracama et al., 2017) in the spectral region from 5.8 to 9.8 ppm, at the cost of suppressing some signals in other regions. The relaxation and acquisition times used allow the complete relaxation of the protons, the signal areas thus being proportional to the number of protons that generate them, except in the suppressed signals, making it possible to use them for quantitative purposes as in previous studies (Guillén & Uriarte, 2012).

References

Ruiz-Aracama, A.; Goicoechea, E.; Guillén, M.D. Direct study of minor extra-virgin olive oil components without any sample modification. ^1H NMR multisupression experiment: A powerful tool. *Food Chem*. **2017**, *228*, 301-314.

Guillén, M.D.; Uriarte, P.S. Monitoring by ^1H nuclear magnetic resonance of the changes in the composition of virgin linseed oil heated at frying temperature. Comparison with the evolution of other edible oils. *Food Control* **2012**, *28*(1), 59-68.

Quantification from ^{1}H NMR spectral data of several compounds present in the starting samples and/or in the lipid extracts of the digestates.

A. Equations used to estimate the molar percentage (%) of the several glyceride structures present in the lipid extract of digestates and the glycerol. In these equations the number of moles (N) of fatty acids and all the glycerides were expressed as follows:

$N_{2\text{-}MG}=Pc*A_K/4$ [eq. S1]

$N_{1\text{-}MG}=Pc*A_L$ [eq. S2]

$N_{1,2\text{-}DG}=Pc*(A_{I+J}-2A_L)/2$ [eq. S3]

$N_{TG}=Pc*(2A_{4.26\text{-}4.38}-A_{I+J}+2A_L)/4$ [eq. S4]

$N_{1,3\text{-}DG}=Pc*(A_{4.04\text{-}4.38}-2A_{4.26\text{-}4.38}-2A_L)/5$ [eq. S5]

$N_{FA}=(Pc*A_F-6N_{TG}-4N_{1,2\text{-}DG}-4N_{1,3\text{-}DG}-2N_{1\text{-}MG}-2N_{2\text{-}MG})/2$ [eq. S6]

$N_{Gol}=(N_{FA}-N_{1,2\text{-}DG}-N_{1,3\text{-}DG}-2N_{2\text{-}MG}-2N_{1\text{-}MG})/3$ [eq. S7]

where Pc is the proportionality constant existing between the area of the ^{1}H NMR signals and the number of protons that generate them, A_K, A_L, A_{I+J} and A_F are the areas of the corresponding signals indicated in Table S2, and $A_{4.26\text{-}4.38}$ and $A_{4.04\text{-}4.38}$ represent the areas of the signals between 4.26 and 4.38 ppm, and between 4.04 and 4.38 ppm, respectively.

Using these equations, the molar percentages of the different kinds of glycerides in relation to the total number of moles of glyceryl structures present (NT_{GS}) were determined as follows:

$NT_{GS}=N_{TG}+N_{1,2\text{-}DG}+N_{1,3\text{-}DG}+N_{2\text{-}MG}+N_{1\text{-}MG}+N_{Gol}$ [eq. S8]

$G\%=100N_G/NT_{GS}$ [eq. S9]

where G is each kind of glyceride (TG, 1,2-DG, 1,3-DG, 2-MG and 1-MG) and N_G the respective number of moles.

$Gol\%=100N_{Gol}/NT_{GS}$ [eq. S10]

B. Estimation of the molar percentages of the main fatty acids (FA) plus acyl groups (AG) (FA+AG). The molar percentages of linolenic (Ln%), linoleic (L%), oleic (O%) and saturated (S%) AG or FA, in relation to the total number of moles of AG plus FA (NT_{AG+FA}) present in the starting oils and in the lipid extracts of the corresponding digestates were estimated as follows:

$Ln\%=100*(A_H/2*A_F)$ [eq. S11]

$L\%=100*(A_G/A_F)$ [eq. S12]

$O\%=U\%–L\%–Ln\%$ [eq. S13]

$S\%=100–U\%$ [eq. S14]

where A_H , A_G A_F are the areas of signals H, G and F indicated in Table S3.

C. Estimation of the concentration of specific compounds in oil samples and in the lipids extract from digestates.

The concentration of the several kinds of specific compounds (X), expressed as micromoles per mole of the sum of AG+FA present, was estimated by using the following equations:

$$[X] = [(A_X/n)/(A_D/2)]*1000 \qquad \text{[eq. S15]}$$

where A_X, is the areas of the signals selected for the quantification of each specific compound (X), present in the oil samples and in the lipid extract from digestates and n the number of protons that generate each signal given in Tables S4 and S5 and A_D is the area of the signal D in Table S3.

Operating Conditions for the SPME-GC/MS Experiments

1. The fiber used for the headspace components extraction was coated with Divinylbenzene/Carboxen/Polydimethylsiloxane (DVB/CAR/PDMS, 50/30 μm film thickness, 1 cm long, acquired from Supelco (Sigma-Aldrich, St. Louis, MO, USA)). It was inserted into the headspace of the sample and maintained for 55 min at 50 ºC, after a pre-equilibration time of 5 min. The fiber containing the components extracted was desorbed for 10 min in the injection port (splitless mode with 5 min purge time) of a 7890A gas chromatograph equipped with a 5975C inert MSD with Triple Axis Detector (Agilent Technologies, Palo Alto, CA, USA) and a computer operating with the ChemStation program. A fused silica capillary column was used (60 m length, 0.25 mm inside diameter, 0.25 μm film thickness; from Agilent Technologies Inc., Palo Alto, CA), coated with a nonpolar stationary phase (HP-5MS, 5% phenyl methyl siloxane). The operation conditions were the following: the injector and interface temperatures were held at 250 °C and 305 °C respectively, and helium at a constant pressure of 117 kPa (16.9 psi) was used as the carrier gas. The oven temperature was initially held at 50°C for 5 min, increased from 50 to 300 °C at a rate of 4 °C/min, and then held at 300 °C for 30 min. Mass spectra were recorded at an ionization energy of 70 eV, with data acquisition in Scan mode. The temperatures of the ion source and the quadrupole mass analyzer were kept at 230 and 150 °C, respectively. A reference sample of known composition was periodically analyzed in order to verify the sensitivity of the SPME-GC/MS experiments as in previous studies (Guillén et al., 2005).

Reference

Guillén, M.D.; Cabo, N.; Ibargoitia, M.L.; Ruiz, A. Study of both sunflower oil and its headspace throughout the oxidation process. Occurrence in the headspace of toxic oxygenated aldehydes. *J. Agric. Food Chem.* **2005**, *53*, 1093–1101.
